# Tracing catchment fine sediment sources using the new SIFT (SedIment Fingerprinting Tool) open source software

**DOI:** 10.1016/j.scitotenv.2018.04.126

**Published:** 2018-09-01

**Authors:** S. Pulley, A.L. Collins

**Affiliations:** Sustainable Agriculture Sciences Department, Rothamsted Research, North Wyke, Okehampton EX20 2SB, UK

**Keywords:** Sediment, Sediment source tracing, Sediment fingerprinting, Catchment management, Uncertainty

## Abstract

The mitigation of diffuse sediment pollution requires reliable provenance information so that measures can be targeted. Sediment source fingerprinting represents one approach for supporting these needs, but recent methodological developments have resulted in an increasing complexity of data processing methods rendering the approach less accessible to non-specialists. A comprehensive new software programme (SIFT; SedIment Fingerprinting Tool) has therefore been developed which guides the user through critical data analysis decisions and automates all calculations. Multiple source group configurations and composite fingerprints are identified and tested using multiple methods of uncertainty analysis. This aims to explore the sediment provenance information provided by the tracers more comprehensively than a single model, and allows for model configurations with high uncertainties to be rejected. This paper provides an overview of its application to an agricultural catchment in the UK to determine if the approach used can provide a reduction in uncertainty and increase in precision. Five source group classifications were used; three formed using a k-means cluster analysis containing 2, 3 and 4 clusters, and two a-priori groups based upon catchment geology. Three different composite fingerprints were used for each classification and bi-plots, range tests, tracer variability ratios and virtual mixtures tested the reliability of each model configuration. Some model configurations performed poorly when apportioning the composition of virtual mixtures, and different model configurations could produce different sediment provenance results despite using composite fingerprints able to discriminate robustly between the source groups. Despite this uncertainty, dominant sediment sources were identified, and those in close proximity to each sediment sampling location were found to be of greatest importance. This new software, by integrating recent methodological developments in tracer data processing, guides users through key steps. Critically, by applying multiple model configurations and uncertainty assessment, it delivers more robust solutions for informing catchment management of the sediment problem than many previously used approaches.

## Highlights

• Sediment source fingerprinting methods have become complex and inaccessible.
• SIFT is comprehensive, user-friendly and free sediment fingerprinting software.
• Multiple source group classifications and composite fingerprints are used.
• Different methods of uncertainty analysis are used to validate each model.
• Localised sources were identified to contribute to channel bed sedimentation.

## Graphical abstract

**Figure:**
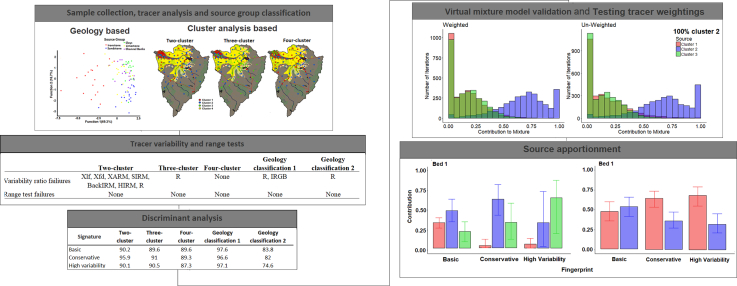

## 1 Introduction

Numerous studies have now used sediment source fingerprinting to investigate specific catchment management problems (Collins et al., 2010a; Gellis and Walling, 2011; Miller et al., 2015; Owens et al., 2017; Collins et al., 2017), yet its application as a standard research tool remains limited. As such, Mukundan et al. (2012) highlighted the need to streamline the sediment fingerprinting approach before it can have wider application as part of a regulatory framework for catchment management issues.

Since the publication by Mukundan et al. (2012), several sediment fingerprinting papers have highlighted uncertainties associated with certain procedural steps, such as tracer conservativeness, tracer corrections, weightings and statistical operations (Koiter et al., 2013; Smith and Blake, 2014; Laceby and Olley, 2015; Pulley et al., 2015a; Laceby et al., 2017; Collins et al., 2017; Owens et al., 2017). This questioning of procedures in the sediment fingerprinting approach is necessary for the science to move forward but it is also necessary to communicate to land managers a streamlined and robust procedure. Collins et al. (2017) recently proposed a methodological decision-tree to aid in the application of sediment fingerprinting for catchment management, which identified the numerous and challenging decisions which must be considered.

One fundamental requirement underpinning successful sediment source fingerprinting is that selected tracers can robustly discriminate between potential sediment sources (Foster and Lees, 2000). It has, however, been shown that simply achieving discrimination in a linear discriminant analysis is not in itself sufficient for reliable source apportionment (Rowan et al., 2000; Sheriff et al., 2015; Pulley et al., 2015a). For example, equifinality problems with source apportionment have long been recognised (Rowan et al., 2000; Small et al., 2002). In addition, tracer concentrations can be controlled by numerous environmental factors, such as geology (Laceby and Olley, 2015), soil type, hydrology and topography (Blundell et al., 2009; Jordanova et al., 2012), anthropogenic pollutants (Foster and Charlesworth, 1996) and land use (Walling et al., 1993), which will often result in high within-source group variabilities if broad source groups such as those based on land use or surface/subsurface sources are used (Pulley et al., 2016). A low between-source group variability in tracer concentrations will also cause tracer non-conservatism to have a larger effect on un-mixing model outputs, as the sediment provenance signal used for discrimination is small (Collins et al., 2010a, Collins et al., 2010b; Sheriff et al., 2015; Pulley et al., 2016).

In response to the need to streamline sediment source fingerprinting data processing for accessible use by end users, a comprehensive new software programme (SIFT; SedIment Fingerprinting Tool) has been developed in R with a user-friendly GUI based around the Shiny package. SIFT guides the user through all critical data analysis decisions without the requirement for specialist knowledge. By way of example, this paper demonstrates the utility of SIFT for applying a combination of multiple different models and uncertainty assessment techniques to the same dataset to produce a more robust interpretation of sediment source fingerprinting results than the use of a single model.

## 2 Study area

**Fig. 1:**
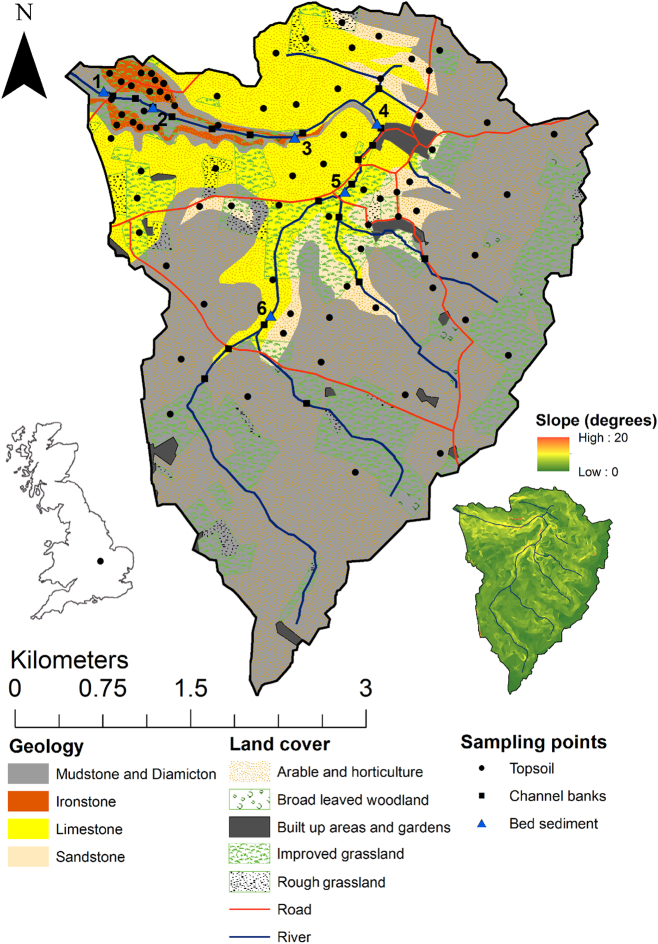
The study catchment and sampling points.

The study used to demonstrate SIFT was conducted in a small tributary of the River Nene in the East Midlands of the UK (Fig. 1). The 15.3 km^2^ catchment was selected based on its heterogenous geology which is likely to exhibit large contrasts in topsoil properties allowing for evaluation of optimum source classification, discrimination and apportionment using SIFT. The lower catchment is composed of outcrops of Jurassic oordial ironstone and Lias mudstone. The middle of the catchment is underlain by Jurassic Blisworth formation and Cornbrash limestone, with Jurassic Kellaways member sandstone at the upper edge of this deposit. The upper catchment is underlain by Quaternary Oadby member diamicton and Jurassic Lias mudstone. The mudstone and diamicton geologies are classified as the same “clays” geology for this study as previous research within the Nene basin found them to be indistinguishable using magnetic, radionuclide and geochemical tracers (Pulley, 2014). Soils in the lower catchment overlying limestone, ironstone and sandstone are freely draining loamy soils, and soils in the upper catchment overlying diamicton are loamy and clayey with poor drainage.

The study area has a mean annual rainfall of 638 mm (Pulley et al., 2015a). Land use is dominated by cultivation with 28% of the catchment used as improved grassland. Most grassland fields showed evidence of having been cultivated in the recent past as part of crop rotation. Much of the area underlain by ironstone is utilised as grassland, unlike those underlain by sandstone, limestone and clays which are mostly cultivated. There is evidence of historical limestone quarrying in the lower catchment with a large number of limestone cobbles present in cultivated fields. The catchment is gently sloping (mean 3.3°) with steeper areas of land close to the river channel (6 to 10°). Land underlain by limestone and ironstone is generally more steeply sloping than sandstone and clays. There are wide (3–5 m) buffer strips of woodland or grassland adjacent to river channels throughout most of the catchment; however, some ditches in cultivated fields had buffers <1 m wide. During the time of sampling (August–September 2016), cultivated soils were dry with deep cracking present. There was no visual evidence of gully or rill erosion within the catchment.

## 3 Methods

### 3.1 Field and laboratory methods

Samples of potential sediment sources were retrieved from four pre-determined groups; ironstone (18 samples), sandstone (20 samples), limestone (20 samples) and clay (mudstone and diamicton) topsoils (20 samples), and channel banks (20 samples) (Fig. 1). Each topsoil sample was a composite of five subsamples collected from within a 3 m radius of the sampling point. Topsoils were collected from the top 2 cm of the soil profile as this is the limiting depth of erosion (Walling and Woodward, 1995). Channel bank samples were collected as a composite of three subsamples from within 2 m of the sampling point. Only the lower 2/3 of the typically ~30 cm high banks was sampled to improve the likelihood of effective discrimination between surface and subsurface sources. All samples were collected using an enamelled stainless-steel trowel.

Samples of sediment deposited on the channel bed were retrieved from five locations (Fig. 1) using the bed disturbance method of Lambert and Walling (1988), recently tested by Duerdoth et al. (2015). These were a composite of three repetitions within a 5 m reach of river channel. It was observed that samples Bed 4 and Bed 5 were from an area of the river bed which experienced significant sedimentation due to river modification and dense aquatic vegetation. These sediments appeared to be stored under anoxic conditions with the potential for processes of dissolution diagenesis to affect their associated tracers.

Source and sediment samples were oven dried at 40 °C and sieved to 38 μm to limit the potential for particle size related uncertainties (Pulley and Rowntree, 2016; Laceby et al., 2017). Organic matter was removed using hydrogen peroxide (H_2_O_2_) treatment (Pulley et al., 2018). Approximately 5 g of sediment was added to 30 ml of 33% H_2_O_2_ and was left at room temperature for 12 h before being heated at 70 °C until dry. The prepared samples were packed into transparent polythene sample bags and scanned to a pdf file using a Cannon MG5600 colour scanner. The images were imported into GIMP 2 open source image editing software and red, green and blue intensities for each sample were recorded on a scale of 0–255 in the RGB colour model. Using these RGB values, the colour indices shown in Supplementary Table 1A were calculated (Viscarra Rossel et al., 2006; Ray et al., 2004). The magnetic signatures shown in Supplementary Table 1B were measured following the methods of Foster et al. (2008). Analytical uncertainties (coefficients of variability) were established as 0.4% (Red), 0.6% (Green) and 0.6% (Blue), together with 0.8% (χlf), 15.59% (χfd), 2.30% χARM, 1.96% SIRM, 3.36% (BackIRM), and 4.72% (HIRM).

### 3.2 The SIFT sediment source tracing data processing methodology

**Fig. 2:**
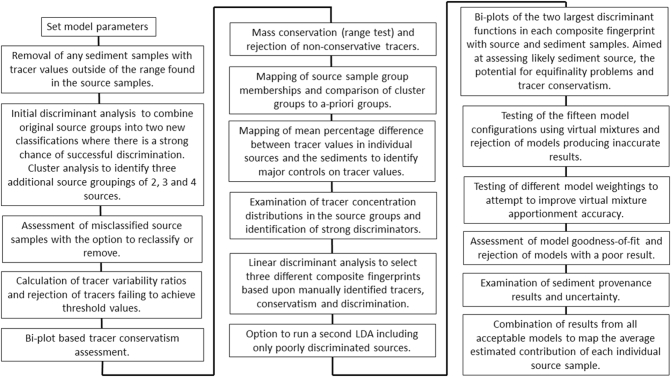
Flow diagram of the stages of the SIFT methodology.

The tracer data processing methodology in SIFT used is loosely based upon the decision-tree recently proposed by Collins et al. (2017), with the aim of producing a robust assessment of uncertainty. Due to the efficiency of the R programming language, the software allows for multiple models with multiple different source group configurations and composite fingerprints to be run together with efficient processing times. Due to this functionality, a composite result comprising multiple model outcomes can be developed, providing greater insight into sediment provenance than the use of a single source group classification method which is common to the majority of studies published to date. The capacity to run multiple un-mixing model structures also allows for model configurations with high associated uncertainties which are assessed using multiple criteria including virtual mixtures to be rejected. The following sections describe each stage of the methodology in the SIFT software and justify their inclusion. Fig. 2 shows a flow diagram of each stage in the SIFT methodology.

#### 3.2.1 Tracer data preparation

##### 3.2.1.1 Removal of any sediment samples with multiple tracer values outside of the range found in the source samples

It is initially tested whether each measured tracer in each sediment sample falls within the full minimum to maximum range of values found in the source samples (Foster and Lees, 2000). This stage of the methodology is aimed at removing any target sediment samples that have likely been heavily affected by tracer non-conservatism. Retaining such samples would likely result in erroneous sediment provenance conclusions.

##### 3.2.1.2 Source group classification

The samples in the initial five geology-based source groups were included in a preliminary linear discriminant analysis (LDA) to gain an indication of the potential for the measured tracers to discriminate between these groups. Discrimination was evaluated using a bi-plot of the two largest discriminant functions and a confusion matrix. Where discrimination was judged to be unsuccessful, two or more source groups were combined into a single group. This stage of the methodology is aimed at limiting the potential for poor source group discrimination to undermine robust source apportionment. Two geology-based source group classifications were manually assigned at this stage in the SIFT methodology; Classification 1, where few source groups were strongly discriminated, and Classification 2, where more source groups were selected, and good discrimination was less certain although still likely.

Three additional source group classifications were generated using a k-means cluster analysis based on the methods of Walling et al. (1993), Walling and Woodward (1995) and Pulley et al. (2016) (Table 1). Solutions with two, three and four source groups were generated by the cluster analysis output, which did not incorporate any a-priori criteria such as land use or geology. The optimal cluster classifications were identified by repeating the cluster analysis five times and selecting the solution with the highest value of the between-cluster sum of squares / the total sum of squares (total within-cluster sum of squares + the between-cluster sum of squares). Where a cluster group was generated containing fewer than five source samples, the analysis was repeated up to five additional times to attempt to identify solutions with more numerically balanced clusters.

**Table 1:** The five source group classifications used.

Classification	Structure
Two-cluster	Each source sample is assigned into one of two clusters according to the results of a k-means cluster analysis.
Three-cluster	Each source sample is assigned into one of three clusters according to the results of a k-means cluster analysis.
Four-cluster	Each source sample is assigned into one of four clusters according to the results of a k-means cluster analysis.
Geology classification 1	The original four geology source groups are combined to produce as many new source groups as possible with the requirement of very strong discrimination between them.
Geology classification 2	The original four geology source groups are combined to produce as many new source groups as possible with the requirement of moderate discrimination between them.

##### 3.2.1.3 Assessment of misclassified source samples

A second LDA including all tracers was then used to identify any source samples which are likely to be misclassified in the two geology-based source classifications. Samples were evaluated using a scatter plot of the two largest discriminant functions, with samples identified as potentially misclassified in the analysis output highlighted in the colour of the source group they are a best fit to. In addition, a map of the catchment with misclassified samples highlighted is produced. Where it is clearly identified that the samples are misclassified for a genuine reason, samples are reclassified into their correct group. Where a sample has outlying tracer values which cannot be attributed to misclassification, it is removed to avoid introducing high within-source variability. Possible genuine reasons for sample misclassifications include: (1) outcrops are small or irregularly shaped causing the geology shown on the map to not reflect that actually present; (2) an underlying rock type covers too small an area to significantly impact the properties of its overlying soils; (3) soil erosion is causing material from upslope to form a blanket over native soils, meaning that the underlying geology is not reflected by the sample collected; (4) if channel banks are shallow and share their properties with surface material, and; (5) if channel bank collapse and slumping causes banks to be composed of displaced surface material. There is no test for outliers in the source groups as the un-mixing modelling approach does not assume a normal distribution, and therefore the potential for outliers to introduce uncertainty is minimised; however, outlying samples can be optionally removed at this point.

#### 3.2.2 Tracer variability ratios and conservatism testing

The ability of the tracers to discriminate robustly between the source groups/clusters comprising each of the five classification schemes was assessed by calculating tracer variability ratios (Pulley et al., 2015a). This represents the ratio of the percentage difference in median tracer concentration between a pair of source groups and the mean of the within-source group coefficient of variation.

$$\;\left(\left(\bar{X}\max-\bar{X}\min\right)/\bar{X}\min\right)/\bar{X}\mathrm{cov}$$

where $\bar{X}$max is the maximum mean tracer concentration of either source group, $\bar{X}$min is the minimum mean tracer concentration of either source group, and $\bar{X}$cov is the mean coefficient of variation for the pair of source groups (calculated as the median absolute deviation divided by the median).

A ratio lower than 1 indicates that the noise of within-source group variability is larger than the between-group signal, which is likely to result in very high uncertainty associated with un-mixing model outputs (Pulley et al., 2015a). For this reason, any tracer with a mean variability ratio of below 1, when considering all pairs of source groups, was removed from further use. In addition, any tracer with a maximum ratio of below 2, when considering all pairs of source groups, was also removed from use to further limit uncertainty in un-mixing model outputs.

##### 3.2.2.1 Bi-plot based conservatism assessment

The first test for tracer non-conservatism is based upon the use of bi-plots (Oldfield and Wu, 2000; Laceby et al., 2015; Pulley et al., 2015b). Pairs of tracers significantly correlated within the source sample dataset with an r^2^ higher than 0.8 are plotted against each other with the sediment samples overlaid onto the same plot. This determines if the relationships between pairs of tracers present in the source samples are maintained in the sediment samples. If a relationship is maintained, it suggests a high degree of tracer conservatism; alternatively, if the relationship is not maintained, tracers in question were removed from further analysis.

##### 3.2.2.2 Range tests and rejection of non-conservative tracers

To further minimise the likelihood of non-conservative tracers being used in the un-mixing model, it is determined if the concentrations of each tracer within the target sediment samples fall within the medians +/− one median absolute deviation (MAD) and the minimum – maximum range of the source groups (Foster and Lees, 2000; Collins et al., 2010a, Collins et al., 2010b; Wilkinson et al., 2013). If each tracer concentration in >40% of the sediment samples fell outside of the median +/− one MAD, and the tracer concentration in 80% of target sediment samples fell outside the full range of the source samples, that tracer was removed from further use. The low 40% threshold was selected to allow some leeway in tracer inclusion as if sediment originates from an area of the catchment with highly distinctive tracer values, then a tracer could fall outside of the median +/− one MAD range whilst still being conservative and a useful discriminator. The 80% threshold was set to allow for one sediment sample to fail the minimum-maximum range test as if one sediment sample is heavily affected by some form of non-conservatism it may be unique to that sample rather than affecting the entire sediment dataset.

#### 3.2.3 Mapping of source and tracer characteristics

##### 3.2.3.1 Mapping source sample group membership

The group/cluster membership of each source sample in each of the five source group classifications is plotted onto a map of the catchment. The three-different cluster analysis derived source groups were compared to catchment geology, land use and topography so that any correspondence of a cluster group with a specific landscape feature could be identified. This information is aimed at aiding the interpretation of the cluster results.

##### 3.2.3.2 Mapping the mean percentage differences between source and sediment sample tracer concentrations

The percentage differences between the concentrations of the tracers in sources and sediments are calculated and mapped to give a preliminary indication of how likely each source sample is to contribute sediment to the river (Pulley et al., 2017). These maps are also used to assess to what extent each of the five source classifications fit the measured tracers. The source and sediment tracer values are normalised to between 0 and 1 by dividing each value by the maximum found for that tracer in the source dataset. The absolute difference between the mean concentration of each tracer in sediment samples and each individual source sample is calculated and expressed as a percentage of the mean concentration in the sediment samples (Pulley et al., 2017). Differences were first calculated as a mean for all tracers, and then were calculated for individual tracers to allow for the identification of any tracers which highlight different source samples to others and may be particularly useful for source discrimination.

#### 3.2.4 Determining and assessing composite fingerprints

##### 3.2.4.1 Source discrimination

Three composite fingerprints were produced for each of the five source classifications to assess variation in sediment provenance estimates dependent on the tracers used (Collins et al., 2012). Rather than basing the different composite fingerprints upon different statistical procedures they were, instead, based upon the conservatism of tracers and their ability to discriminate the sources in question. Each of the three composite fingerprints was determined using a stepwise LDA to identify the composite fingerprint best able to discriminate between the source groups for each of the five source classification schemes (Collins et al., 1997). In the stepwise process, a 0.1% improvement in overall discrimination was required for an additional tracer to be included in the composite fingerprints. Each stepwise LDA was repeated three times and the solution able to achieve the highest discrimination was retained for use in the un-mixing model. If composite fingerprints were formed containing fewer than two tracers, the LDA was repeated to attempt to determine a fingerprint containing a greater number of tracers, as lower uncertainties have been shown to be associated with larger fingerprints (Small et al., 2002; Sheriff et al., 2015). After performing the initial LDA the option exists to run a second LDA including only poorly discriminated source groups to attempt to improve overall discrimination. In the case of the study dataset however, this stage did not provide any additional benefit. Different tracers were forced into the LDA solution to form the three different composite fingerprints for each source group classification.

(i) The first fingerprint is a basic fingerprint selected using the LDA output, but with an option to include additional tracers via manual forcing. Forced inclusion is based upon an examination of the differences between sources and sediments maps, and by examining plots of the percentile distributions of each tracer in the source groups. Any tracer which is particularly effective at discriminating a specific source group can be forced into the composite fingerprint.
(ii) A “conservative” fingerprint is formed by forcing in any tracers which passed the bi-plot conservatism test. This composite fingerprint aims to capture the tracers most likely to be unaffected by organic matter, particle size and diagenesis effects.
(iii) A “high variability” fingerprint is formed by forcing in the tracers with the highest variability ratios to attempt to achieve the lowest range of uncertainty in model outputs and limit the potential for uncertainties associated with sediment delivery from only a small area of the catchment which may have outlying tracer values. In this case study, one tracer was forced into the high variability fingerprint for each source group present, starting with the highest ratio tracer and working down a ranked list.

##### 3.2.4.2 Bi-plots of sources and sediments

Bi-plots of the two largest discriminant functions generated by the LDAs were produced for each of the three composite fingerprints for each of the five source classifications. Samples belonging to each of the source groups were colour coded and the DF scores for the sediment samples were calculated and included in the plot. These plots provide an indication of sediment provenance which can be compared to the un-mixing model results as a form of model validation.

#### 3.2.5 Un-mixing modelling

The un-mixing model is based upon a modified version of the frequentist model developed by Collins et al. (1997), with Monte Carlo uncertainty analysis (Rowan et al., 2000) (assistance with successfully coding the model in r was given by Gorman-Sanisaca et al., 2017). Prior to un-mixing, all source and sediment tracer concentrations are rescaled between 0 and 1 by dividing by the maximum value found in the source samples for each tracer. No data corrections for organic matter and particle size were used in the model as the sample preparation in this study was designed to limit the potential for these uncertainties. The model was also modified so that when two tracers in a source group were significantly correlated with an r^2^ > 0.8 the same correlation was maintained in the generated Monte Carlo random numbers (Laceby and Olley, 2015). The Monte Carlo iterations also produce random numbers following the same distribution as the tracer concentrations in the sampled source groups (Pulley et al., 2017), rather than assuming a normal distribution or using location and scale estimators (Rousseeuw and Croux, 1993) such as median and median absolute deviation. Specifically, for each tracer ~5% of the Monte Carlo iterations fell between the 0th and 5th percentile values in each source group, ~5% from the 5th–10th percentile values, etc. This sampling method is included because using a distribution which is not representative of the real distribution of tracer concentrations in the source group is potentially a major source of uncertainty. No weightings for organic matter and particle size were used as the sample preparation method was aimed at limiting the potential for these uncertainties.

The unmixing-model was run for 3000 model iterations; for each iteration, the goodness-of-fit (GOF) was calculated as the root mean square of relative errors between the modelled and the actual sediment tracer properties (Motha et al., 2003). Any model iteration with a GOF below 0.35 was rejected as a non-viable solution. The percentage of model iterations passing this GOF threshold and their mean GOF were recorded for model evaluation. The 0.35 limit was selected through preliminary trials on the dataset as a higher threshold often resulted in no model iterations passing for some samples.

#### 3.2.6 Model testing and weighting

##### 3.2.6.1 Testing of the model configurations using virtual mixtures

Virtual sample mixtures consisting of the tracer concentrations of hypothetical mixtures of different sources which have been mathematically calculated also represent a means to assess if the general modelling approach is likely to deliver an acceptably low range of uncertainty. Equifinality problems with source apportionment (Rowan et al., 2000; Small et al., 2002), the effects of sediment delivery from only a small part of a catchment, the effects of a high within source group variability, or the potential impact of tracer non-conservatism can all be assessed using these mixtures.

The tracer values of virtual mixtures were calculated to evaluate model accuracy and uncertainty (Lees, 1997; Franks and Rowan, 2000; Haddadchi et al., 2014; Palazón et al., 2015). These consisted of 100% contributions from each source group (the source group median), equal proportions of each source group (the mean of the source group medians) and equal proportions of each source group but with a randomly selected percentile value (0th–100th in intervals of 5%) for each tracer from each source group. The un-mixed composition of the virtual mixtures was compared to their actual composition to determine if source apportionment was accurate (the dominant sediment source was correctly identified). Models failing to achieve this were discarded.

##### 3.2.6.2 Testing of model weightings to improve virtual mixture apportionment accuracy

The virtual mixtures can be used to trial a variety of weightings to determine if they improved source apportionment accuracy. The first weighting trialled was a variability ratio weighting, calculated by dividing the mean variability ratio for each tracer by the largest mean variability ratio for any tracer within the composite fingerprint. This ratio was aimed at representing a combination of the discriminatory efficiency and within-source group variability weightings developed by Collins et al. (2010b). The second set of weightings trialled were manually selected based upon three criteria. Firstly, the tracers most strongly correlated with the second discriminant function for each composite fingerprint were weighted as these were often important discriminators between poorly discriminated sources, but only represented a small percentage (<20%) of total discriminatory power. Therefore, these weightings were aimed at compensating for the dominating effects of tracers which are only able to discriminate easily separated source groups. Secondly, the percentile distributions of each tracer in each source group classification were examined to identify the tracers best able to discriminate between poorly differentiated source groups. The order of highest – lowest concentration of each tracer in the source groups was particularly noted, as if the order is the same for all tracers, problems of equifinality are likely to be present when un-mixing sediment provenance. Thirdly, the mapped differences between sources and sediments for each tracer were used to identify the tracers best able to identify sediment contributions from specific sources or areas of the catchment. Each tracer selected was given 3× its normal weighting in the un-mixing model. The option exists in the software to trial multiple weightings and selects the set which perform optimally. Each source group classification and composite fingerprint was examined separately, and weightings were only used for those models where it showed a clear improvement in source apportionment precision and accuracy.

##### 3.2.6.3 Assessment of model goodness-of-fit

The sediment samples were run through the un-mixing model with each of the five source group classifications and three different composite fingerprints which produced an acceptable result for the virtual mixtures. Weightings were applied where they were shown to improve source apportionment. Any model where all of the Monte Carlo iterations fell below the 0.35 threshold was judged to have been unsuccessful and so was rejected.

#### 3.2.7 Sediment provenance results

##### 3.2.7.1 Presentation of sediment provenance results

The median, 25th and 75th percentile proportions are presented for each sediment sample for each source classification and composite fingerprint to summarise the un-mixing model results. These results were interpreted in the context of any sources of uncertainty identified in previous methodological steps and to determine if the results conform to what might be expected with the spatial distribution of the source groups and the observed catchment characteristics.

##### 3.2.7.2 Combination of all model results to map the likely contribution of each source sample to sediment provenance

The average percentage contribution of each source sample to each sediment sample is calculated from every model producing an acceptable result when apportioning the composition of the virtual mixtures, and an acceptable GOF. These maps provide a simple yet detailed visualisation of the probable sources of each sediment sample.

## 4 Results

### 4.1 Sediment sample screening and source group classification

Tracer concentrations in sediment samples Bed 1, 2, 4 and 6 fell within the full minimum to maximum range found in the source groups, and as such these samples were retained for further analysis. For sample Bed 5, R, G, HRGB and IRGB fell outside of the range of values found in the source samples, and for Bed 3 BackIRM fell outside of this range, indicating the non-conservatism of these tracers in these samples. However, as most of the measured tracers fell within the range of the source groups, these samples were retained for further analysis.

**Fig. 3:**
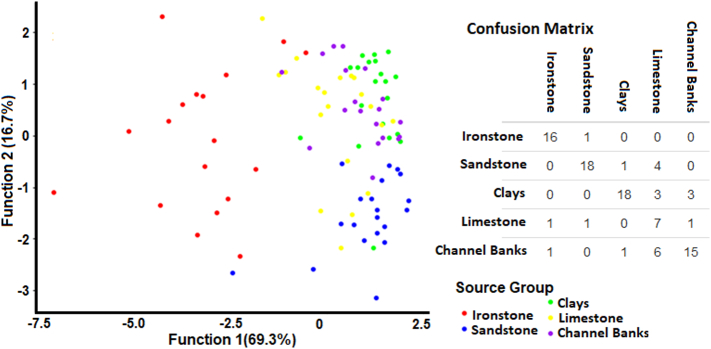
Bi-plot of the two largest discriminant functions generated by the initial LDA of the five geology-based source groups, with confusion matrix.

The initial LDA identified that the two largest discriminant functions (DFs) were responsible for 86% of source discrimination (Fig. 3). DF1 primarily provided discrimination between Ironstone topsoils and the other sources. Sandstone topsoils were reasonably discriminated by DF2 but, overlapped slightly with other sources. Discrimination between limestone topsoils, clay topsoils and channel banks was minimal. As such, the first geology-based source group classification aimed at achieving the best discrimination possible was:

Group 1: Ironstone and Group 2: Sandstone, Limestone, Clays and Channel Banks.

The second geology-based source classification with a greater number of individual groups comprised:

Group 1: Ironstone, Group 2: Sandstone, and Group 3: Limestone, Clays and Channel Banks.

**Fig. 4:**
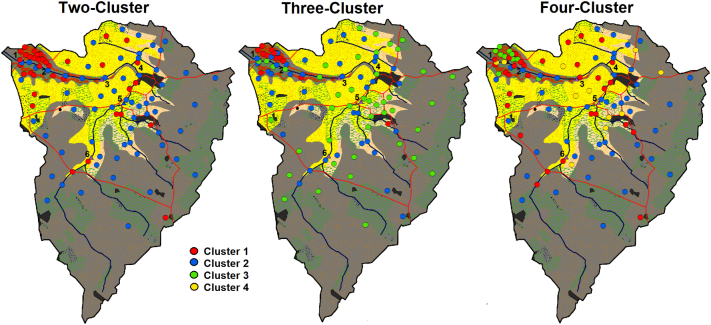
The mapped cluster analysis based sediment source classifications.

In all three-cluster analysis derived source classifications, ironstone topsoils were mostly concentrated within the membership of one particular cluster (clusters 1, 1 and 3; Fig. 4). However, this cluster also contains some samples from the middle catchment in the two-cluster solution, and in all three cluster derived classifications some samples originally classified as ironstone are not included. In addition to the ironstone dominated cluster, the three-cluster solution divides the middle and upper catchment into two sources which appear unrelated to geology. However, it is of note that cluster 2 contains most channel bank samples in the lower half of the catchment and cluster 3 contains most channel bank samples in the upper catchment. The four-cluster classification is similar to the three-cluster solution; however, it identifies an additional small source cluster with its samples primarily located in the centre of the catchment.

### 4.2 Misclassified samples

**Fig. 5:**
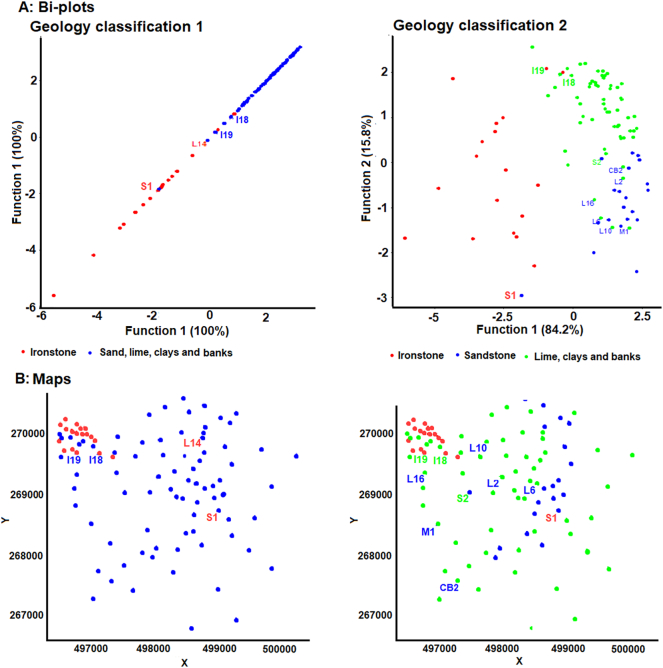
Bi-plots (A) and maps (B) of potentially misclassified source samples as identified by the LDA. A labelled sample was potentially misclassified. The label colour identifies the source group the sample is a better fit in.

Sample S1 (sandstone) was identified as potentially misclassified and was a better fit to the ironstone source group in both of the geology-based source classifications (Fig. 5). This sample was, however, distant from areas of ironstone within the catchment, so it was removed from further use. In both source classifications, the Ironstone samples I18 and I19 were identified as a better fit as sandstone, clay or limestone samples. As these samples were on the boundary of the two geologies, and likely represented topsoil properties not reflecting the ironstone bedrock shown on the geology map, both were reclassified. There were a number of potentially misclassified samples between the sandstone group and clays, limestone and channel banks group in geology classification 2. These were, however, judged to reflect poor source discrimination and therefore these were not reclassified.

### 4.3 Summary statistics and variability ratios

Mean variability ratios for all pairs of all tracers were 2.1 for the two-cluster classification, 3.8 for the three-cluster classification, 4.6 for the four-cluster classification, 3.1 for geology classification 1 and 2.5 for geology classification 2 (Table 2). Both two source group solutions had low variability ratios with the geology-based Classification 1 performing slightly better than the two-cluster classification. Geology classification 2 also had low variability ratios, which likely reflected the limited discrimination between the sandstone group and the clay, limestone and channel banks group. Most tracers passed the required mean ratio of 1 and maximum ratio of 2 thresholds in all five source classifications.

**Table 2:** Median, median absolute deviation, mean and maximum variability ratios for all pair combinations of source groups; ratios in bold pass the threshold values for further inclusion in the apportionment modelling.

Two-cluster																
Median	χlf	χfd	χARM	SIRM	BackIRM	HIRM	R	G	B	HRGB	IRGB	SRGB	SI	HI	CI	RI
Cluster 1 (35 samples)	0.72	43.57	6.32	7.33	6.01	0.68	182.1	147.1	124.4	2.98	151.73	28.75	0.19	4.03	0.11	0.84
Cluster 2 (64 samples)	0.34	16.85	2.73	4.08	3.09	0.49	187.3	158.2	135.05	1.26	160.22	24.85	0.16	3.46	0.08	0.64
Median absolute deviation																
Cluster 1	0.53	46.23	6.35	6.03	5.32	0.38	4	3.11	3.11	0.37	2.77	2.59	0.02	0.2	0.01	0.05
Cluster 2	0.19	13.64	2.28	2.32	2.12	0.18	5.93	4.89	4.74	0.78	4.87	2.52	0.01	0.27	0.01	0.07
Mean variability ratio	**1.7**	**1.7**	**1.43**	**1.15**	**1.2**	0.8	**1.06**	**2.9**	**2.85**	**3.66**	**2.3**	**1.64**	**2.38**	**2.59**	**2.83**	**3.8**
Max variability ratio	1.7	1.7	1.43	1.15	1.2	0.8	1.06	**2.9**	**2.85**	**3.66**	**2.3**	1.64	**2.38**	**2.59**	**2.83**	**3.8**


Three-cluster																
Median	Xlf	xfd	XARM	SIRM	BackIRM	HIRM	R	G	B	HRGB	IRGB	SRGB	SI	HI	CI	RI
Cluster 1 (11 samples)	1.35	108.71	14.46	16.64	13.85	1.39	183	145.4	121.2	3.13	149.53	31.4	0.2	4.05	0.11	0.89
Cluster 2 (38 samples)	0.45	22.9	3.28	5	4	0.52	182.3	149.2	127.1	2.62	152.67	28	0.18	3.91	0.1	0.78
Cluster 3 (50 samples)	0.34	16.59	2.73	4.01	3.09	0.5	187.15	160.8	137.8	1.06	161.95	24.7	0.15	3.36	0.08	0.62
Median absolute deviation																
Cluster 1	0.36	24.56	7.21	4.08	2.47	0.33	3.71	5.11	2.82	0.72	4.47	1.19	0.01	0.29	0	0.05
Cluster 2	0.3	21.8	2.96	3.38	2.91	0.21	5.93	5.04	5.19	0.52	4.15	1.26	0.01	0.23	0.01	0.09
Cluster 3	0.16	11.78	1.95	2.23	1.89	0.17	5.49	5.34	4.45	0.54	5.31	1.74	0.01	0.17	0.01	0.04
Mean variability ratio	**4.69**	**7.04**	**4.39**	**4.86**	**5.43**	**3.95**	0.64	**2.08**	**2.96**	**3.88**	**1.82**	**3.55**	**4.13**	**2.33**	**5.54**	**4.02**
Max variability ratio	**7.89**	**11.86**	**7.08**	**7.86**	**8.81**	**6.24**	0.92	**3.1**	**4.93**	**5.27**	**2.65**	**5.01**	**6.07**	**3.37**	**8.22**	**7.06**


Four-cluster																
Median	Xlf	Xfd	XARM	SIRM	BackIRM	HIRM	R	G	B	HRGB	IRGB	SRGB	SI	HI	CI	RI
Cluster 1 (31 samples)	0.54	28.37	3.8	5.35	4.24	0.54	182.1	148.2	125.5	2.8	151.77	28	0.18	4	0.1	0.81
Cluster 2 (47 samples)	0.3	14.8	2.55	3.89	2.9	0.46	187.1	157.6	134.9	1.3	159.9	25.2	0.16	3.47	0.08	0.65
Cluster 3 (10 samples)	1.41	112.15	15.42	16.72	13.85	1.41	182.45	143.7	121.2	3.21	148.43	31.1	0.2	4.16	0.11	0.89
Cluster 4 (11 samples)	0.57	35.1	6.03	5.71	4.6	0.57	189.7	165.9	144.5	0.58	166.8	22.2	0.13	3.21	0.07	0.55
Median absolute deviation																
Cluster 1	0.29	23.71	3.67	3.18	2.76	0.22	4.45	3.11	2.97	0.37	2.72	1.26	0.01	0.2	0.01	0.05
Cluster 2	0.14	10.21	1.97	2.04	1.61	0.16	5.93	3.26	3.41	0.59	3.11	1.33	0.01	0.2	0.01	0.04
Cluster 3	0.34	16.59	4.08	4.08	2.47	0.3	4.23	5.26	3.85	0.72	5.54	1.45	0.01	0.24	0.01	0.06
Cluster 4	0.32	26.51	4.65	1.71	1.65	0.11	3.26	2.67	3.85	0.3	2.37	3.85	0.02	0.13	0	0.02
Mean Variability Ratio	**4.68**	**7.07**	**4.6**	**4.67**	**5.41**	**4.11**	**1.09**	**3.87**	**4.04**	**8.26**	**3.39**	**3.03**	**3.99**	**3.45**	**6.33**	**6.58**
Max Variability Ratio	**10.46**	**15.71**	**9.75**	**10.42**	**11.88**	**9.25**	**2.01**	**6.64**	**6.57**	**12.39**	**6.3**	**5.35**	**5.98**	**6.05**	**11.6**	**12.54**


Geology classification 1																
Median	Xlf	Xfd	XARM	SIRM	BackIRM	HIRM	R	G	B	HRGB	IRGB	SRGB	SI	HI	CI	RI
Ironstone	1.18	102.32	13.64	13.15	10.94	1.18	183	145.4	121.2	2.94	149.53	31.28	0.2	4.01	0.11	0.89
Sand, lime, clay and banks	0.36	17.64	2.73	4.47	3.36	0.5	184.7	156.75	134.05	1.61	158.67	25.67	0.16	3.57	0.08	0.67
Median absolute deviation																
Ironstone	0.55	66.32	8.74	5.97	4.72	0.38	4.52	6.15	4.97	0.52	4.65	1.37	0.01	0.21	0.01	0.06
Sand, lime, clay and banks	0.21	14.12	2.26	2.73	2.25	0.18	5.93	7.04	6.75	1.02	7.02	3.04	0.02	0.34	0.02	0.08
Mean variability ratio	**4.34**	**6.63**	**5.45**	**3.65**	**4.1**	**3.96**	0.33	**1.79**	**2.32**	**2.03**	**1.62**	**2.69**	**3.2**	**1.65**	**2.77**	**3.52**
Max variability ratio	**4.34**	**6.63**	**5.45**	**3.65**	**4.1**	**3.96**	0.33	1.79	**2.32**	**2.03**	1.62	**2.69**	**3.2**	1.65	**2.77**	**3.52**

Geology classification 2																

Median	Xlf	Xfd	XARM	SIRM	BackIRM	HIRM	R	G	B	HRGB	IRGB	SRGB	SI	HI	CI	RI
Ironstone	1.18	102.32	13.64	13.15	10.94	1.18	183	145.4	121.2	2.94	149.53	31.28	0.2	4.01	0.11	0.89
Sandstone	0.37	19.58	3.49	4.75	3.59	0.57	190.4	161.4	138.7	1.65	162.97	27.45	0.16	3.56	0.09	0.62
Lime, clay and banks	0.35	16.73	2.46	4.06	3.07	0.46	183.3	154.9	132.2	1.57	157.33	25.65	0.16	3.59	0.08	0.68
Median absolute deviation																
Ironstone	0.55	66.32	8.74	5.97	4.72	0.38	4.52	6.15	4.97	0.52	4.65	1.37	0.01	0.21	0.01	0.06
Sandstone	0.11	10.83	1.54	2.15	1.83	0.13	4	4.15	6.82	0.7	3.31	2.74	0.02	0.18	0.02	0.09
Lime, clay and banks	0.23	16.82	1.97	2.65	2.27	0.15	5.63	8.01	7.12	1.19	6.47	2.52	0.02	0.41	0.02	0.11
Mean variability ratio	**3.44**	**4.74**	**4.61**	**2.83**	**3.1**	**3.43**	**1.11**	**1.93**	**2.05**	**1.53**	**2.06**	**1.96**	**2.18**	**1.3**	**1.96**	**2.4**
Max variability ratio	**5.93**	**7.81**	**6.86**	**4.14**	**4.64**	**4.98**	1.77	**3.23**	**3.2**	**2.65**	**3.5**	**3.09**	**3.34**	**2.4**	**2.94**	**3.65**

Bold values signify values exceeding the threshold of 1 for the mean variability ratio and 2 for the maximum variability ratio.

### 4.4 Bi-plot conservatism testing

**Fig. 6:**
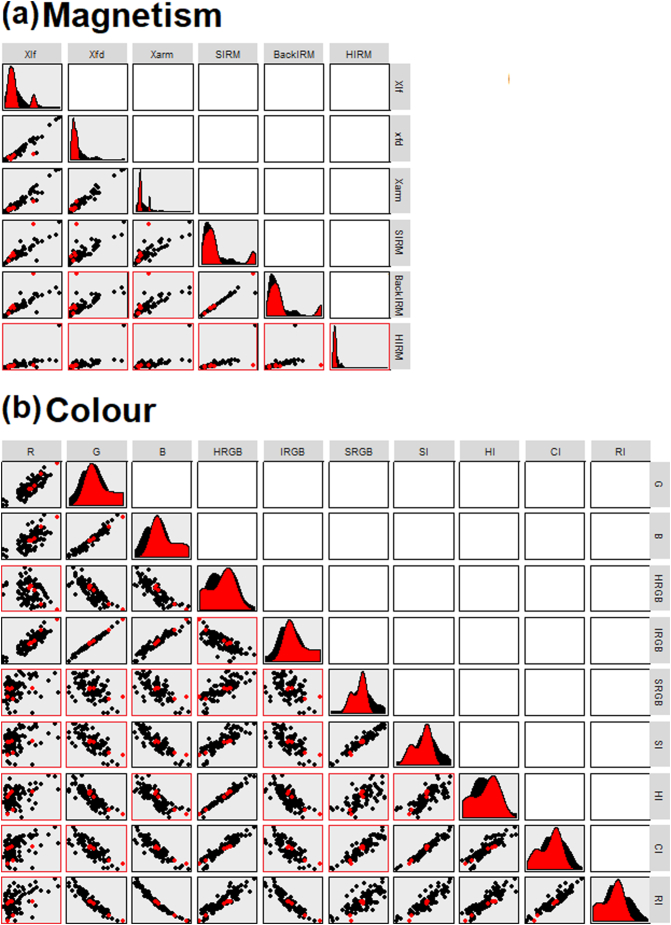
Bi-plots of source (black) and sediment (red) samples, plots bordered in red failed to achieve the required correlation coefficient for progressing in the analysis.

Most of the mineral magnetic tracers were significantly correlated with each other (*p* < 0.05; r^2^ > 0.8), and most of the colour traces were also significantly correlated (Fig. 6). There were no significant correlations between individual magnetic and colour tracers. Most tracers in the sediments followed the relationships observed in the sources apart from samples Bed 3 and 5. Sample Bed 5 was previously identified as having a number of colour tracers which fell outside of the minimum-maximum range found in the source samples, and SIRM and IRM-100 in sample Bed 3 were high. Therefore, it is likely that most of the tracers used are conservative in four of the six samples but the results for samples Bed 3 and 5 should be carefully evaluated to identify if they are likely to be reliable.

### 4.5 Range test

All tracers passed the range test in all source groups by exceeding the 40% of sediment samples falling between the highest source group median + one MAD to the lowest source group median – one MAD range for each tracer (Table 3). Colour tracers again performed poorly with sample Bed 5, and magnetic tracers performed poorly with sample Bed 3. The magnetic tracers χlf, χfd, χARM as well as SRGB, performed best with a total of 97–100% of sediment samples falling within the required range. Blue, SRGB and HRGB performed worst with only 70.2% of samples falling within the required ranges. Tracer values in most sediment samples fell within the full minimum – maximum range in the source groups. Exceptions were BackIRM for sample Bed 3, and R, G, HRGB, IRGB for sample Bed 5. The 80% pass rate threshold was exceeded by all tracers.

**Table 3:** The percentage of sediment samples falling within the maximum + one MAD to minimum − one MAD range of values for each tracer in the source classifications.

	χlf	χfd	χARM	SIRM	BackIRM	HIRM	R	G	B	HRGB	IRGB	SRGB	SI	HI	CI	RI
Two-cluster	83	100	100	83	83	83	83	67	67	83	67	100	67	83	67	67
Three-cluster	100	100	100	83	83	83	83	67	67	83	67	100	67	83	67	67
Four-cluster	100	83	100	83	83	83	83	83	83	83	83	100	100	83	83	83
Geology classification 1	100	100	100	83	83	83	67	67	67	67	67	100	67	67	83	67
Geology classification 2	100	100	100	83	83	83	83	67	67	83	67	83	67	83	67	83
Percent within minimum - maximum	100	100	100	100	83	100	83	83	100	83	83	100	100	100	100	100

### 4.6 Mapped differences between sources and sediments

**Fig. 7:**
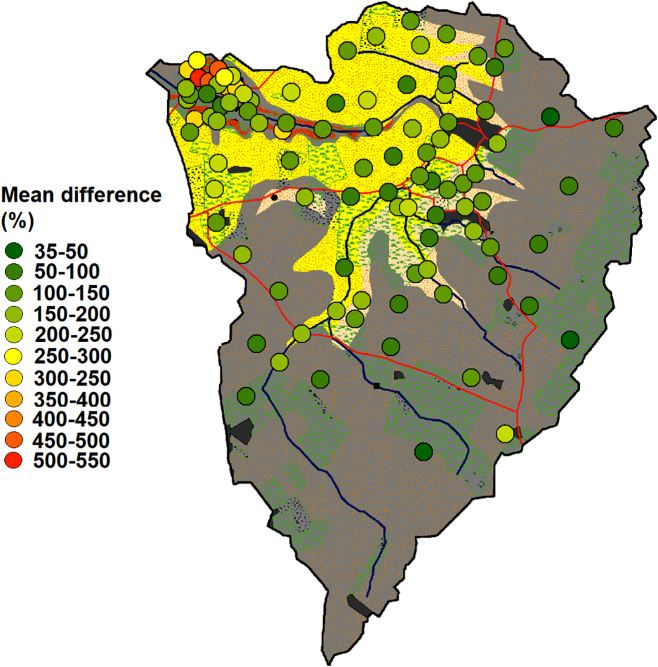
Mean percentage differences between each source sample and the mean of all sediment samples for all tracers.

Mapping the mean percentage difference between all tracer concentrations of each source sample and the mean for all sediment samples identified that the ironstone source samples in the lower catchment are very dissimilar to the sampled sediments (Fig. 7). The samples in the middle and upper catchment and the channel bank samples in the lower catchment have the most comparable properties to the sampled sediments. However, there is some variability within the middle to upper catchment, with some samples being more dissimilar to the sediments than others.

**Fig. 8:**
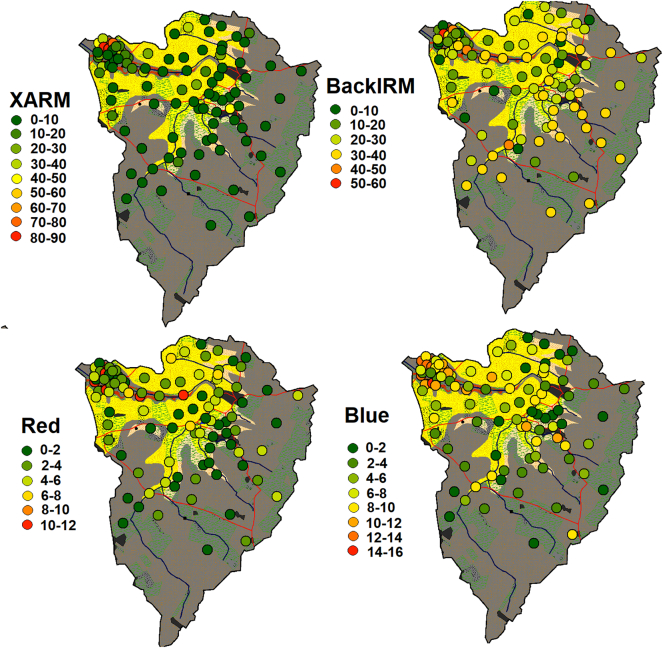
Mean percentage differences between each source sample and the mean of all sediment samples for individual tracers.

When examining individual tracers, χARM was most effective at isolating ironstone source samples but showed little variation in the source samples retrieved from the middle and upper catchment (Fig. 8). BackIRM has more variability in the middle and upper catchment and is therefore likely to discriminate sources other than ironstone topsoils. Similarly, Blue is able to differentiate between samples throughout the entire catchment, but with a different trend to χARM and with smaller percentage differences. Unlike the other tracers, Red is comparable in almost all sources and sediments explaining its poor variability ratios. The other tracers showed comparable trends to one of the four examples presented.

### 4.7 Source discrimination

**Fig. S1:**
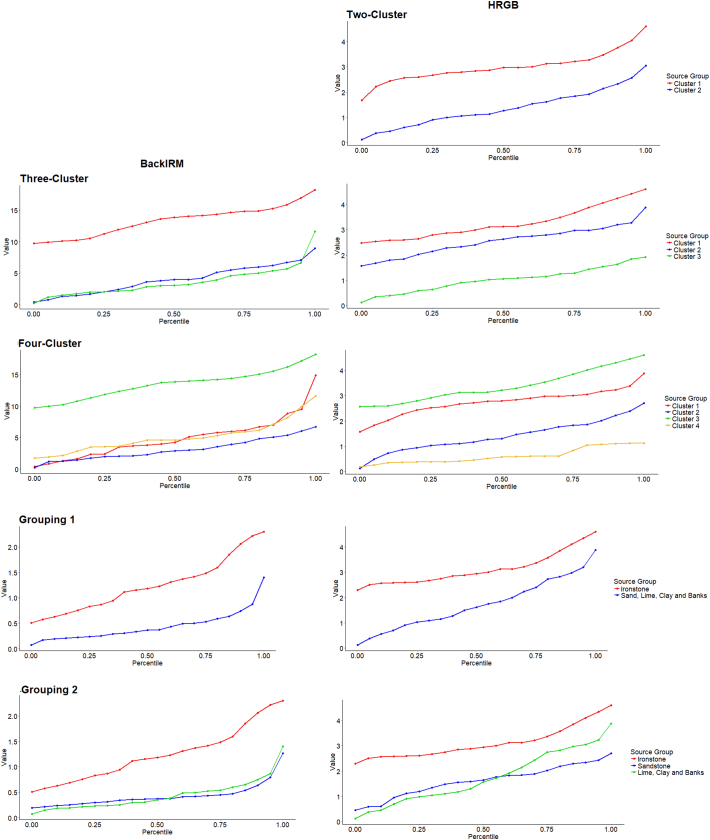
Percentile distributions of tracer concentrations in the source groups.

There were generally strong correlations between all colour as well as all magnetic tracers, resulting in comparable percentile distributions for each tracer type (Fig. S1). Therefore, only results for one magnetic and one colour tracer are presented. With the mineral magnetic tracers, there was generally a large difference between source groups/clusters representing ironstone and the other source groups, but non-ironstone sources were poorly separated. Colour tracers separated the non-ironstone sources more effectively; however, all tracers placed the source groups into the same highest to lowest order, suggesting that problems of equifinality may be present in the final outputs. SIRM and BackIRM were forced into the basic LDA composite fingerprints for three and four cluster source classifications, as an initial run of the SIFT software identified problems associated with equifinality were present where magnetic tracers were under represented. HRGB was also forced into each composite fingerprint as the best colour discriminator.

The stepwise LDA identified composite fingerprints able to achieve good source discrimination (>80%) for most of the five source classifications and composite fingerprint types (Table 4). The one poor discriminator was the high variability fingerprint for source Classification 2. It is of note that only colour tracers passed the range test for the two-cluster classification and therefore no magnetic tracers are present in its composite fingerprints (Table 5). As all tracers passed the bi-plot conservatism test all tracers were included in the conservative fingerprints making these the largest (Table 5).

**Table 4:** The percentage of source samples correctly classified into their respective groups by optimum composite fingerprints selected by the stepwise LDA.

Signature	Two-cluster	Three-cluster	Four-cluster	Geology classification 1	Geology classification 2
Basic	90.2	89.6	89.6	97.6	83.8
Conservative	95.9	91	89.3	96.6	82
High variability	90.1	90.5	87.3	97.1	74.6

**Table 5:** The optimum composite fingerprints identified by the LDA.

Basic	
Two-cluster	B, HI, RI
Three-cluster	HI, SIRM, BackIRM, G, HRGB
Four-cluster	RI, SIRM, BackIRM, G, HRGB, R
Geology classification 1	SIRM, BackIRM, HRGB, SI, CI
Geology classification 2	B, HI, RI, SIRM, BackIRM, G, HRGB, SI, CI, χlf, χfd, χARM, SRGB


Conservative	
Two-cluster	B, HI, RI, G, HRGB, SI, CI, IRGB
Three-cluster	B, HI, RI, SIRM, BackIRM, G, HRGB, SI, CI, χlf, χfd, χARM, SRGB, IRGB, HIRM
Four-cluster	B, HI, RI, SIRM, BackIRM, G, HRGB, R, SI, CI, χlf, χfd, χARM, SRGB, IRGB, HIRM
Geology classification 1	B, RI, SIRM, BackIRM, HRGB, SI, CI, χlf, χfd, χARM, SRGB, HIRM
Geology classification 2	B, HI, RI, SIRM, BackIRM, G, HRGB, SI, CI, χlf, χfd, χARM, SRGB, IRGB, HIRM


High variability	
Two-cluster	B, RI, HRGB
Three-cluster	RI, BackIRM, G, CI, χlf, χfd
Four-cluster	B, RI, BackIRM, G, HRGB, χfd
Geology classification 1	BackIRM, SI, χfd, χARM
Geology classification 2	B, HI, RI, SIRM, BackIRM, χlf, χfd, χARM

### 4.8 Bi-plots of sources and sediments

**Fig. 9:**
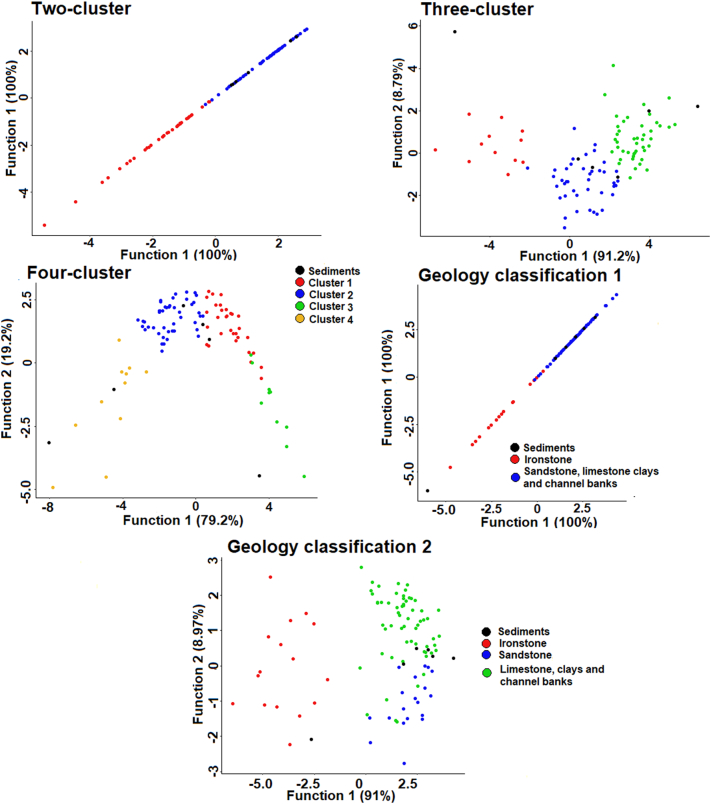
Bi-plots of the two largest discriminant functions for the source groups and sediment samples with the final composite fingerprints for each source classification.

**Fig. S2:**
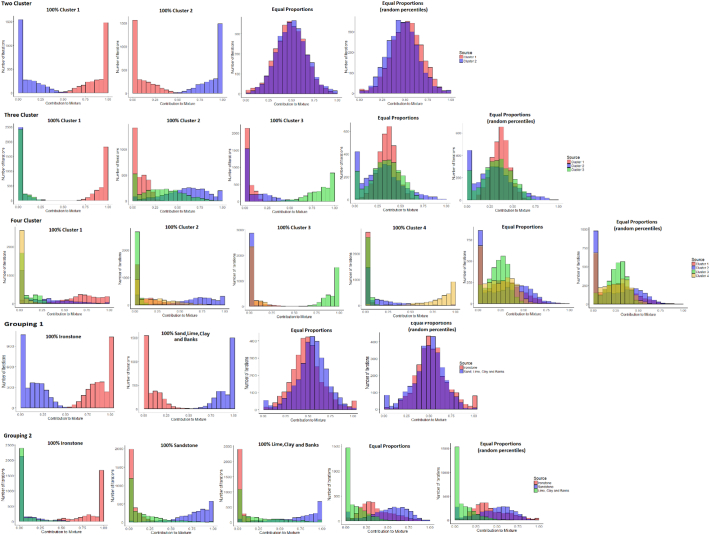
Bi-plots of the two largest discriminant functions for the source groups and sediment samples with the final composite fingerprints for each source classification.

Only plots for the basic composite fingerprints are shown as all three fingerprints generally produced a similar plot for each source classification (Fig. 9); however, all plots are provided in the online Supplementary information (Fig. S2). As only two source groups are present in the two-cluster and Geology classification 1 there is only a single DF. For the other classifications the two largest DFs are shown.

The two-cluster classification plot indicates that Cluster 2, which is primarily composed of non-ironstone sources, is likely to dominate contributions to the bed sediment samples. Reasonable discrimination between the two sources is present, with only one source sample overlapping the two clusters. The three-cluster solution also shows good discrimination. DF1 linearly discriminates between all three clusters and DF2 provides the separation of clusters 1 and 3, and cluster 2, which is necessary to avoid equifinality related uncertainties. However, DF2 represents only 8.79–9.13% of the total discriminatory power. A mixture of clusters 2 and 3 likely dominates contributions to three of the sediment samples, and cluster 3 appears to dominate contributions to two samples. For the four-cluster solution, clusters 1 and 2 appear to dominate contributions to three samples, and inputs from cluster 4 dominate contributions to two of the samples. DF1 is again able to discriminate linearly between the four clusters. DF2 representing 20% of total discrimination, discriminates clusters 1 and 4 from clusters 2 and 3. Discrimination between clusters 2 and 3 is limited to a small amount by DF1, suggesting that apportionment of contributions from these sources may have high associated uncertainties.

Geology classification 1, with two source groups, shows good discrimination and sediment provenance dominated by non-ironstone sources. Geology classification 2 shows notably poorer discrimination between the source groups when compared to the other source classifications. Discrimination between the sandstone topsoil and limestone, clays and channel banks group is poor, and is only provided by DF2, which accounts for ~5% of total discriminatory power. A mixture between sandstone soils and limestone, clays and channel banks appears to dominate contributions to the sediment. Samples Bed 3 and Bed 5 fall outside of the range of the source samples in some plots, confirming the non-conservatism of magnetic and colour tracers in these samples.

### 4.9 Virtual mixtures

**Fig. S3:**
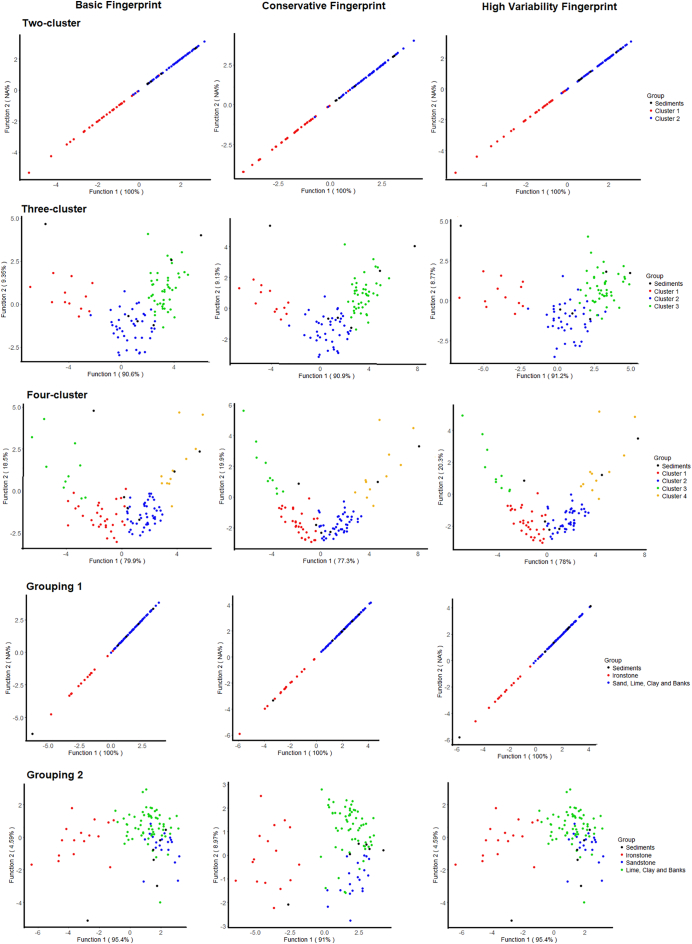
Probability density functions of the virtual mixtures.

Virtual mixture apportionment with the two-cluster classification produced the correct composition of the mixtures (Fig. S3). Uncertainties for the mixtures of 100% of each cluster were low; however, with the equal proportions of each cluster they were high. The artificial mixtures consisting of random percentiles overestimated contributions from cluster 2 by 10–20% (Fig. S3). Source apportionment for the three-cluster solution was again generally accurate but with a higher associated range of uncertainty. Uncertainty was especially high when apportioning a 100% contribution from cluster 2, with significant estimated contributions from cluster 3. The mixtures of equal proportions of each cluster were again generally accurately apportioned, but with slight (~5%) over and underestimated contributions from clusters 2 and 3, depending upon which of the three fingerprints were used (Fig. S3).

With the four-cluster classification, the un-mixing model correctly identified contributions from clusters 3 and 4. However, when apportioning contributions from clusters 1 and 2, uncertainties were high with significant overlap between the probability density functions (pdfs) for the two sources. It is therefore apparent that the three-cluster solution is the optimum for this specific dataset, as the four-cluster solution starts to exceed the discriminatory ability of the tracers. For the geology-based classifications, Classification 1 produced comparable results to the two-cluster groups but with a higher range of uncertainty. Source apportionment with all three fingerprints for geology-based Classification 2 was unsuccessful. A 100% contribution from clays, limestone and channel banks was not represented the un-mixing model results and equal proportions of the mixtures produced an output heavily biased towards high sandstone topsoil contributions.

### 4.10 Weightings

The tracer variability ratio weighting had little effect on the virtual mixture model pdf outputs and therefore was not used for sediment source apportionment (Table 6). The manual weightings based upon the tracers most strongly correlated with DF2, the percentile distributions of each tracer in each source group classification and the mapped differences between sources and sediments were more effective (Table 6).

**Table 6:** The results of the manually selected 3× weightings on virtual mixture source apportionment.

	Tracers weighted	Improved basic fingerprint apportionment	Improved conservative fingerprint apportionment	Improved high variability fingerprint apportionment
Two-cluster	RI	Yes	Yes	Yes
Three-cluster	HRGB, CI	Yes	Yes	No
Four-cluster	χARM, BackIRM	No	No	No
Geology classification 1	BackIRM	Yes	Yes	No
Geology classification 2	BackIRM, G	No	–	–

### 4.11 Goodness-of-fit

For the cluster analysis derived source classifications, >50% of model iterations exceeded the 0.35 GOF threshold (Fig. S5). The exception was sample Bed 5, where in all but four of the models run, all iterations failed to achieve a GOF higher than 0.35 and therefore were rejected. The mean GOF of the model iterations passing the threshold was high (>0.75). Only the basic Geology classification 2 fingerprint produced a poorer (~0.6) result. Due to the poor performance of Geology classification 2, its results were not considered for further analysis.

### 4.12 Sediment provenance

**Fig. 10:**
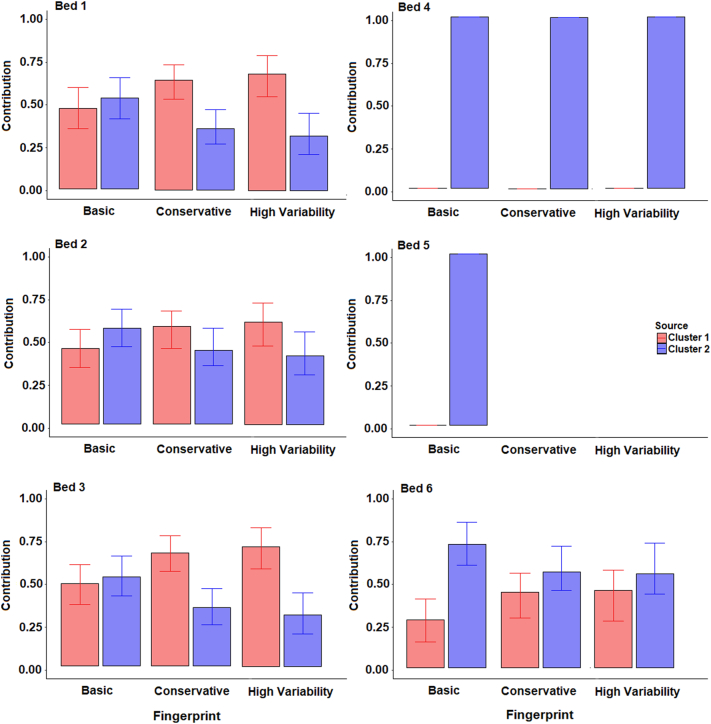
Estimated bed sediment provenance using the two-cluster source classification; median with 25th and 75th percentile uncertainties.

The apportionment results (Fig. 10) produced for the two-cluster source classifications suggested comparable contributions from cluster 1 and cluster 2 for samples in the lower half of the catchment (Bed 1–3), suggesting that localised topsoil inputs from cluster 1 are of importance here. Cluster 1 is far less important in the upper catchment samples (4–6), which is likely due to the lack of area covered by this cluster in the upper catchment. The high cluster 1 contributions to sample Bed 6 suggest significant channel bank inputs. All three composite fingerprints produced similar results although contributions varied by ~20%.

**Fig. 11:**
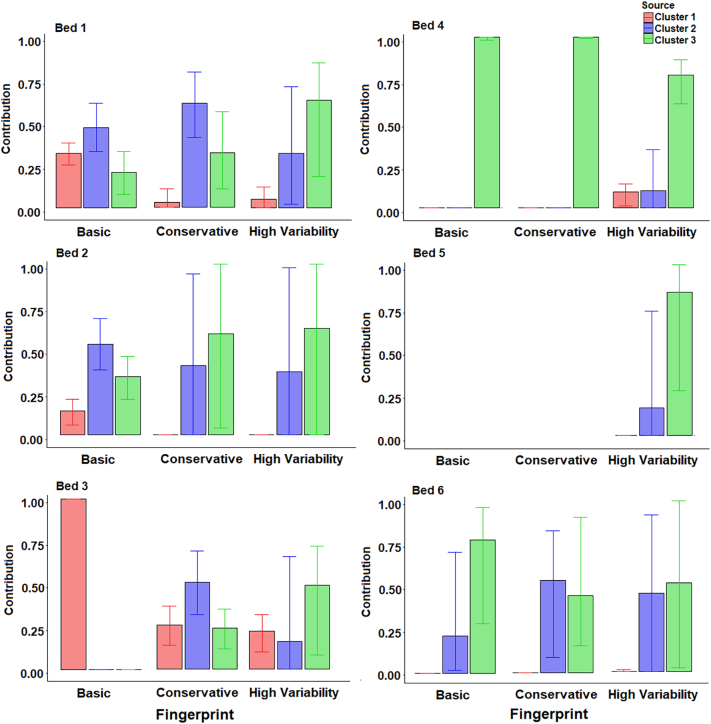
Estimated bed sediment provenance using the three-cluster source groups; median with 25th and 75th percentile uncertainties.

When using the three-cluster classification, the uncertainties associated with conservative and high variability fingerprints were high for sample Bed 2 (Fig. 11). This was primarily because of the sediment sample falling between the two sources in the bi-plots, meaning that multiple model solutions could be ‘mathematically correct’. As such, the basic fingerprint is likely the only result presented that provides information on the provenance of this sample. The presence of χlf, χfd, χARM and BackIRM in the conservative and high variability fingerprints, but not basic fingerprints, is the likely cause of this high uncertainty as these tracers have no ability to discriminate between clusters 2 and 3 and likely dilute the more effective colour tracers.

Cluster 1 represents primarily Ironstone samples in the lower catchment and all models suggest low contributions from this source. The highest contributions from ironstone samples are to sample Bed 3 suggesting that localised inputs from ironstone outcrops in channel banks or topsoil close to this sampling location many be of importance. This finding builds upon that identified for the two-cluster analysis, by suggesting that topsoil inputs in the lower catchment are primarily from soils which are not over the ironstone geology. There is a large discrepancy between the results for sample Bed 3 with the three composite fingerprints. The basic fingerprint estimated a higher contribution from cluster 1 than the other fingerprints. This is likely due to the probable non-conservatism of magnetic tracers (SIRM and BackIRM). Sample Bed 4 is entirely dominated by cluster 2 inputs. No result was produced for sample 5 other than using the high variability fingerprint resulted in very high uncertainty. For sample Bed 6, cluster 2 is predominantly made up of channel bank samples and cluster 3 is composed of topsoils; therefore, the results suggest comparable inputs from both sources.

**Fig. 12:**
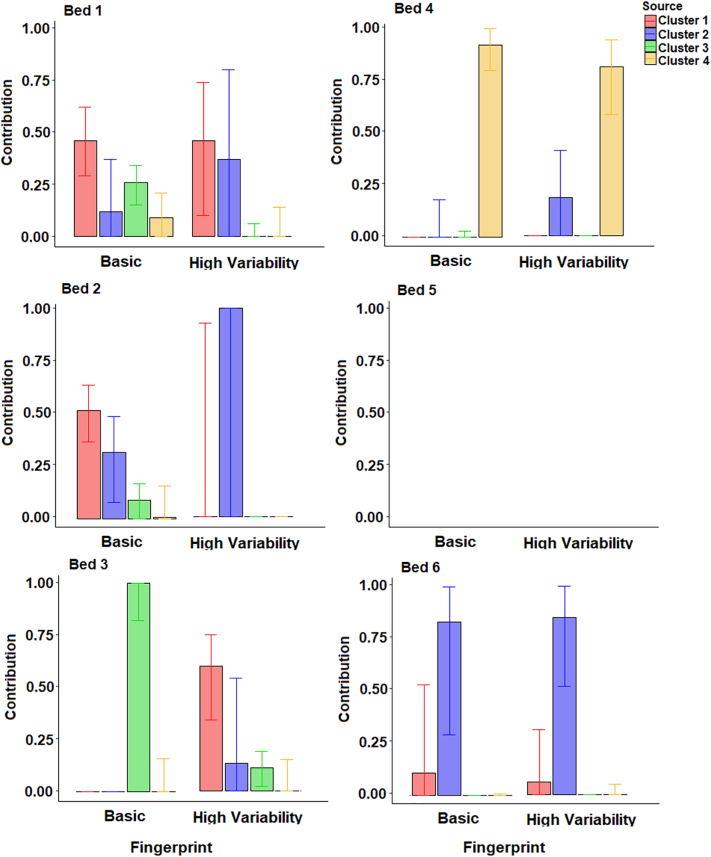
Estimated bed sediment provenance using the four-cluster source groups; median with 25th and 75th uncertainties.

For the four-cluster source classifications (Fig. 12), there were some large discrepancies between the results of the two composite fingerprints which passed the virtual mixture and GOF tests. For sample Bed 1, the basic fingerprint estimated largest contributions from cluster 1 and small contributions from the other clusters, and the high variability fingerprint identified equal contributions from clusters 1 and 2 and little contribution from clusters 3 and 4, albeit with a high range of uncertainty. The one result with an acceptable range of uncertainty for sample Bed 2 (basic fingerprint) identified a similar provenance to sample Bed 1, with clusters 1 and 2 dominating. As with the three cluster classifications (Fig. 11), this suggests little inputs from ironstone topsoils which dominate the membership of cluster 3.

**Fig. 13:**
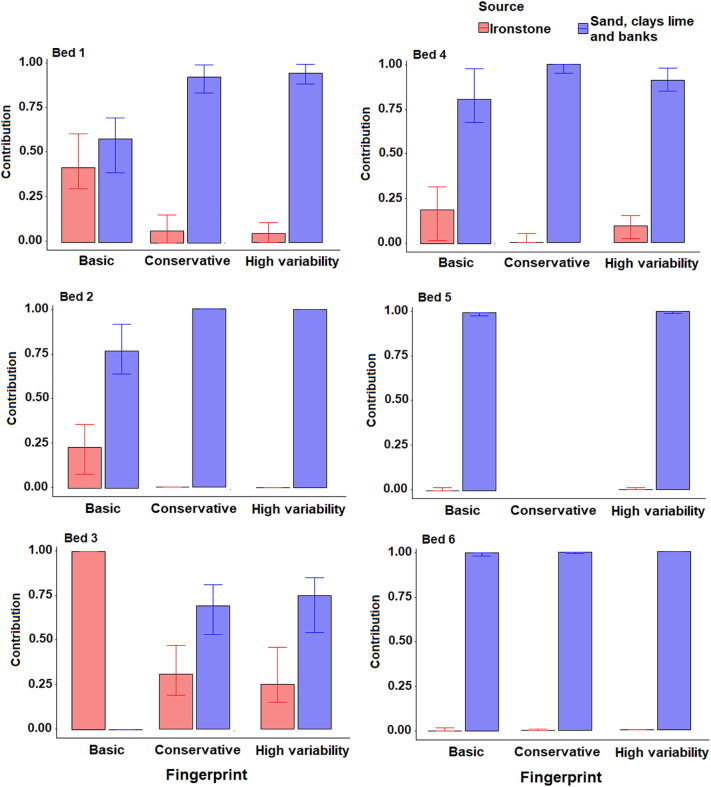
Estimated bed sediment provenance using the Classification 1 source groups; median with 25th and 75th uncertainties.

For Geology classification 1 (Fig. 13), the conservative and high variability fingerprints estimated a higher ironstone contribution when compared to the basic fingerprint. The non-ironstone source group dominated contributions to all sediment samples for the conservative and high variability fingerprints, with only a small ironstone contribution to sample bed 3. The dominance of ironstone contributions to sample Bed 3 when using the basic composite fingerprint is likely caused by the non-conservative SIRM and BackIRM making up 40% of the tracers used.

### 4.13 Mapped sediment provenance

**Fig. 14:**
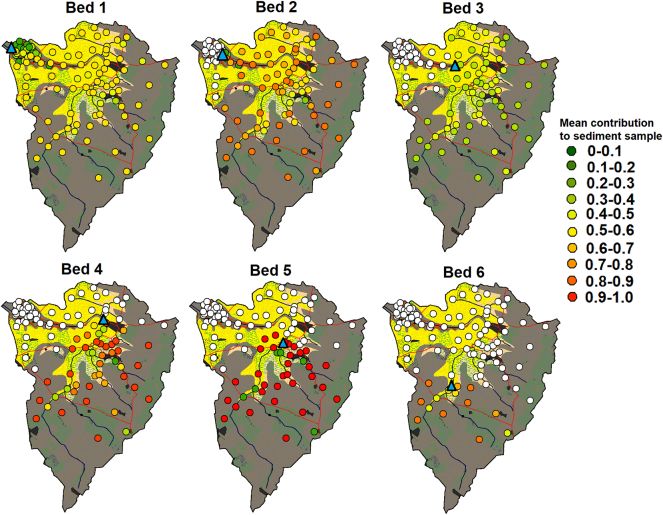
Mapped mean contribution of each source sample to the bed sediment samples predicted by the un-mixing models.

The maps of the combined median contribution to the sediment samples estimated by the reliable models (Fig. 14) provide a qualitative summary of the overall results. The results for the conservative and high variability fingerprints for the four-cluster solution, the basic Geology classification 1 fingerprint and all Geology classification 2 fingerprints were not included in this summary as their associated uncertainties were judged to be too high. For sample Bed 1, low contributions from ironstone samples and comparable contributions from elsewhere in the catchment were estimated. The results for sample Bed 2, suggested roughly equal inputs from across the catchment, apart from nearby ironstone but with a smaller input from a patch of samples in the centre of the catchment over the sandstone geology. Sample Bed 3, was estimated to have low contributions from the entire catchment; although as previously identified, SIRM and BackIRM are likely non-conservative, and therefore the map may not accurately reflect actual sediment provenance. It is also possible that highly localised ironstone inputs dominate contributions to this sample causing the abnormality in tracer concentrations. Samples Bed 4 and 5 suggested that topsoil sources dominate in the upper catchment; however, high contributions are also likely from a few channel bank samples. The results for sample Bed 6 suggested contributions from both banks and surface sources with slightly higher inputs from surface sources. Table 7 summarises the key results from each section.

**Table 7:** A summary of key results.

	Two-cluster	Three-cluster	Four-cluster	Geology classification 1	Geology classification 2
Sediment sample screening	For sample Bed 5, R, G, HRGB and IRGB fell outside of the range of values found in the source samples. For sample Bed 3 Back IRM fell outside of this range.				
Source group classification	Cluster 1: Predominantly contained lower catchment topsoils, Cluster 2: Lower catchment channel banks and upper catchment topsoils.	Cluster 1: Predominantly ironstone samples, Clusters 2 and 3: divide the middle and upper catchment into two sources which appear unrelated to geology but appear spatially grouped.	Comparable to the three-cluster solution, however, it identified an additional cluster of only eight samples with its samples primarily located in the centre of the catchment.	Group 1: Ironstone and Group 2: Sandstone, Limestone, Clays and Channel Banks.	Group 1: Ironstone, Group 2: Sandstone, and Group 3: Limestone, Clays and Channel Banks.
Misclassified samples				Sample S1 (sandstone) was identified as potentially misclassified and was a better fit to the ironstone group so was deleted as it did not fall close to the area of the catchment over ironstone, the Ironstone samples I18 and I19 were also identified as potentially misclassified and fit better as sandstone, clay or limestone samples and reclassified as they were close to the boundary of two geologies	
Mean variability ratios	2.1	3.8	4.6	3.1	2.5
Maximum variability ratio	HRGB, 3.7	χlfd, 11.86	χlfd, 15.71	χlfd, 6.63	χlfd, 7.81
Tracers failing to achieve the variability ratio threshold values	χlf, χlfd, χlARM, SIRM, BackIRM, HIRM, R	R	None	R, IRGB	R
Bi-plot conservatism testing	For sample Bed 5 most colour tracers fall outside of the relationships found in the source samples. For sample Bed 3 SIRM and BackIRM fell outside of the relationships in the source samples.				
Range test	All tracers passed the range test for source classifications by tracer values in 40% of sediment samples falling within the median +/− one MAD range of the source groups and in 80% of sediment samples falling within the minimum to maximum range of the sources.				
Mapped differences between source and sediment tracer concentrations	Ironstone source samples in the lower catchment are very dissimilar to the mean tracer values of the sampled sediments, BackIRM has more variability in the middle and upper catchment whilst XARM shows little variability, Blue is able to differentiate between samples throughout the entire catchment, but with a different trend to χARM				
Distributions of tracers in source groups	With the mineral magnetic tracers there was a large difference between the percentile distribution of values in the source groups/clusters representing ironstone and the other source groups. In contrast non-ironstone sources were poorly separated. Colour tracers separated the non‑ironstone sources more effectively; however, all tracers placed the source groups into the same highest to lowest value order, suggesting that problems of equifinality may be present in model outputs when a large number of source groups are used.				
Source discrimination (percent correctly classified) (basic, conservative, high variability fingerprints)	90.2%, 95.9%, 90.1% (only contains colour tracers)	89.6%, 91%, 90.5%	89.6%, 89.3%, 87.3%	97.6%, 96.6%, 97.1%	83.8%, 82%, 74.6%
Bi-plots of sources and sediments	Cluster 2 likely dominates contributions to the bed sediment, discrimination appears good.	A combination of clusters 2 and 3 likely dominates contributions to three of the sediment samples and cluster 3 appears to dominate contributions to two samples. Discrimination is good however, discrimination between clusters 2 and 3 is only achieved using DF2, which represents 8.79–9.13% of the total discriminatory power	Clusters 1 and 2 appear to dominate contributions to three samples and inputs from cluster 4 dominate contributions to two of the samples. DF2 representing 20% of total discrimination, is able to discriminate clusters 1 and 4 from clusters 2 and 3. Discrimination between clusters 2 and 3 is limited to a small amount by DF1, therefore equifinality related uncertainties are likely in model outputs.	Sediment provenance is dominated by non‑ironstone sources and source discrimination is good.	Ironstone contributes significantly to one sediment sample. The other sediment samples are likely composed of a combination of sandstone, limestone clays and channel banks. Discrimination between ironstone topsoils and other sources is good, discrimination between the sandstone topsoil and limestone, clays and channel banks group is poor and is only provided by DF2, which accounts for ~5% of total discriminatory power.
Virtual mixture source apportionment	Un-mixing models produced the correct provenance of the virtual mixtures. Uncertainties for the mixtures of 100% of each cluster were low; however, with the equal proportions of each cluster they were high.	Mixture apportionment was generally accurate but with a higher associated range of uncertainty than the two-cluster classification. Uncertainty was especially high when apportioning a 100% contribution from cluster 2, with significant estimated contributions from cluster 3 present.	The un-mixing models correctly identified contributions from clusters 3 and 4. However, when apportioning contributions from clusters 1 and 2 uncertainties were high, with significant overlap between the probability density functions for the two sources. The conservative fingerprint failed to identify Cluster 1 as the dominant source when 100% of the mixture was composed of this cluster.	Produced comparable results to the two-cluster groups but with a higher range of uncertainty.	Source apportionment with all three fingerprints for geology-based Classification 2 was unsuccessful. A 100% contribution from clays, limestone and channel banks was not represented in the un-mixing model results and a mixture of equal proportions of the sources produced an output heavily biased towards high sandstone topsoil contributions.
Weightings	A weighting of RI increased the accuracy of mixture apportionment for all three fingerprints.	A weighting of HRGB and CI improved mixture apportionment with the Basic and Conservative fingerprints.	No composite fingerprint improved mixture apportionment. Use of the Conservative fingerprint was discontinued due to its poor performance.	A weighting of BackIRM increased the accuracy of mixture apportionment for the Basic and Conservative fingerprints.	No composite fingerprint improved mixture apportionment. Due to the poor performance of Classification 2, its results were not considered for further analysis.
Goodness of fit	For the cluster analysis derived source classifications, >50% of model iterations exceeded the 0.35 GOF threshold. With the exception of those for sample Bed 5 where in all but four of the models run all iterations failed to achieve a GOF higher than 0.35 and therefore were rejected. The mean GOF of the model iterations passing the threshold was high (>0.75). GOF for geology classification 1 was generally lower than for the cluster-based classifications, the conservative fingerprint for sample Bed 5 had no iterations which exceeded the 0.35 threshold.				
Sediment provenance	For sediment samples in the lower half of the catchment and sample Bed 6 in the upper catchment similar contributions were estimated to originate from cluster 1 and cluster 2. Cluster 2 dominated contributions to samples Bed 4 and 5 in the middle catchment. All three composite fingerprints produced similar results although contributions varied by ~20%.	Contributions from cluster 1 are low in all models apart from sample Bed 3 with the basic fingerprint. Topsoil inputs in the lower catchment are primarily from areas which are not over the ironstone geology. Sediment contributions to sample Bed 3 likely originate from localised channel bank inputs. Bed 3 basic fingerprint estimates a much higher contribution from cluster 1 than the other fingerprints, but consistency is reasonable for all other samples. Uncertainties associated with conservative and high variability fingerprints were high for sample Bed 2. Both clusters 2 and 3 are important sediment sources.	There were some large discrepancies between the results of the two composite fingerprints used. Clusters 2 and 3 appear to dominate contributions in to samples Bed 1, however, the basic fingerprint estimated high contributions from cluster 1. For samples Bed 2, 3, and 5 there was either very poor consistency between the composite fingerprints or no model with an acceptable GOF could be produced. For sample Bed 4 cluster 4 which covers a small area in the centre of the catchment dominates contributions, and for Bed 6 cluster 2 dominates.	Ironstone topsoils a minor source in all but sample Bed 3. The basic fingerprint estimates a larger contribution from ironstone than the other fingerprints.	No result produced

## 5 Discussion

Numerous uncertainties were associated with this sediment fingerprinting study and these are common to applications of the approach. However, by incorporating within SIFT the use of conservatism tests, multiple source group classifications, virtual mixtures, bi-plots of source and sediment samples, multiple composite fingerprints and data visualisation, the modelling results can be interpreted in context of these uncertainties.

When considering the use of multiple different source group classifications, the a-priori source groups proved of variable use as part of this study. Using Geology classification 1, it was determined that ironstone topsoils were minor sediment sources; however, one composite fingerprint suggested higher contributions than the others adding uncertainty to the overall result. Geology classification 1 also only had ironstone and non-ironstone sources as different groups, meaning that results do not provide relevant information about source provenance in the upper catchment where ironstone was not present. Geology classification 2 failed to produce models able to apportion accurately the composition of the virtual mixtures, despite the LDA producing theoretically viable fingerprints. Poor discrimination between all non-ironstone sources using the available tracers was the cause of this result. The cluster analysis derived source groups also produced variable results. As with geology-based Classification 1, the two-cluster classification separated ironstone (cluster 1) from non-ironstone sources (cluster 2), but with some added and subtracted samples from each group. The results of the un-mixing models were more constant as within-source group variability was lower. The consistency may, however, have been due to all magnetic tracers failing to achieve a maximum variability ratio >2, resulting in only colour tracers being used.

The bi-plot and range tests indicated that both colour and magnetic tracers were non-conservative in some sediment samples. The use of H_2_O_2_ and the tracing of a narrow particle size range were aimed at reducing the potential for organic matter and particle size related uncertainties but may have been insufficient to achieve this aim. It was also observed that vegetation within the channel trapped large quantities of sediment in anoxic conditions which may have caused the dissolution of minerals within the sediment, producing a source of uncertainty the sample preparation methodology was unable to account for. The use of the bi-plot and range tests were therefore important so that the tracing results could be interpreted in the context of these uncertainties.

The three-cluster Classification appears to be the optimal combination of highest variability ratios, accurate apportionment of the virtual mixtures, high model GOF, and consistency between the results of different composite fingerprints, whilst dividing the catchment into source groups sufficiently spatially-explicit for catchment management purposes which were predominantly ironstone toposils (cluster 1) and spatially distinct patches of topsoils and channel banks (clusters 2 and 3). The four-cluster source groups resulted in lower variability ratios than the three-cluster solution and, as a result, a larger range of uncertainty in virtual mixture and sediment source apportionment and poorer consistency between the results derived using different composite fingerprints was encountered. As the magnetic and colour tracers used were mostly correlated with other tracers of the same type, only two major discriminant functions were present using the available tracers and, as a result, it is not surprising that effective source discrimination was limited to three source groups. This was likely a result of equifinality where multiple model solutions are mathematically correct using the limited DFs (Rowan et al., 2000). The four-cluster groups were also difficult to interpret for catchment management purposes with only cluster 1 (predominantly ironstone) representing a distinct catchment characteristic, although there was some spatial grouping of source samples belonging to the other three clusters.

A limitation with the cluster analysis derived source groups was that the sampling campaign did not effectively cover the entire catchment at a high resolution. Therefore, there is some difficulty in interpreting which areas of the catchment should be classified into each cluster group so that catchment management interventions can be targeted. A stratified sampling campaign which ensures all areas of the catchment are sampled would be required to overcome this limitation. Whilst this is achievable in small catchments, in larger basins, such a sampling scheme would require significantly more source samples to be collected than when representing sources by land use or geology.

The identification of misclassified source samples as an initial stage of the methodology appears a useful addition when a-priori source groups are used, allowing for an increase in discrimination and decrease in within-source variability. However, the need for personal judgement as to which samples are misclassified potentially introduces uncertainty if poorly discriminated samples are mistakenly misclassified. Therefore, it is recommended that reclassification only be used where robust justification exists for each specific sample. Scale dependency can be a large source of uncertainty in predicted source apportionment and is likely to depend on the specific nature of sediment delivery and the variability in source properties in a catchment (Biddulph et al., 2017). Sample Bed 3 may possibly have originated primarily from only a local source such as an ironstone outcrop in the channel banks, explaining its very high magnetic properties and highlighting the need to examine point source samples close to the retrieved sediment samples. The measured χlf (1.26) and SIRM (28.79) of sample Bed 3 are comparable to the ironstone topsoil samples reported by Pulley (2014); χlf (2.63) SIRM (27.77). Sample 5 was identified by the mass conservation tests as having non-conservative colour. It is likely that reducing anoxic conditions were present on the bed causing the dissolution of the light absorbing minerals in the sediment. The methodology used was not able to mitigate these changes but could limit the potential for erroneous conclusions to be derived using this sample.

The current SIFT software is only useful with discrete tracers. Some researchers have used alternative spectra-based fingerprints. Poulenard et al. (2009), for example, used infrared spectrometry and partial least squares regression, whereby the whole spectrum was used instead of selecting discrete tracers. For NIR spectra to be used in SIFT individual discrete peaks must be identified (Collins et al., 2013).

Overall, the sources of bed sediment appear localised to each bed sediment sampling location. Therefore, the results of this work would best be combined with an analysis of the quantities of bed sediment along reaches to identify where bed sedimentation is most substantial so that management can be targeted accordingly. It remains important to include multiple channel sampling locations in a source fingerprinting study as a means of addressing the scale dependency problem. This result suggests that sediment is likely deposited onto channel beds during periods of low flow from well-connected sources, rather than being deposited during the falling limb of a high flow event following mobilisation and delivery from distal sources.

## 6 Conclusions

This trial of the multiple unmixing model and uncertainty assessment approach used in the SIFT software identified that some un-mixing model configurations produced highly uncertain results, and therefore, its use of multiple different model configurations and assessments may be essential to produce robust results for some datasets. The current version of SIFT can be accessed from the Rothamnsted Research website at: www.rothamsted.ac.uk/facilities-and-resources#DATAREPOSITORIESMODELSANDSOFTWARE-3. An image of each page of SIFT (v1.0) is provided in the online supplementary material.

The following are the supplementary data related to this article.

**Fig. S4:**
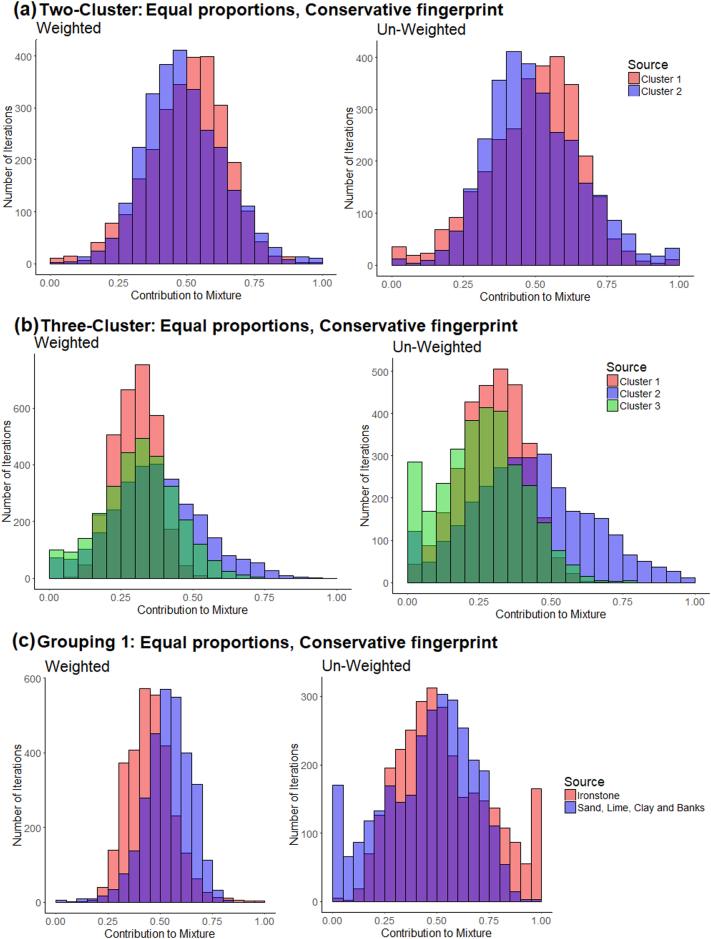
Selected pdfs for virtual mixtures run through weighted and unweighted un-mixing models.

**Fig. S5:**
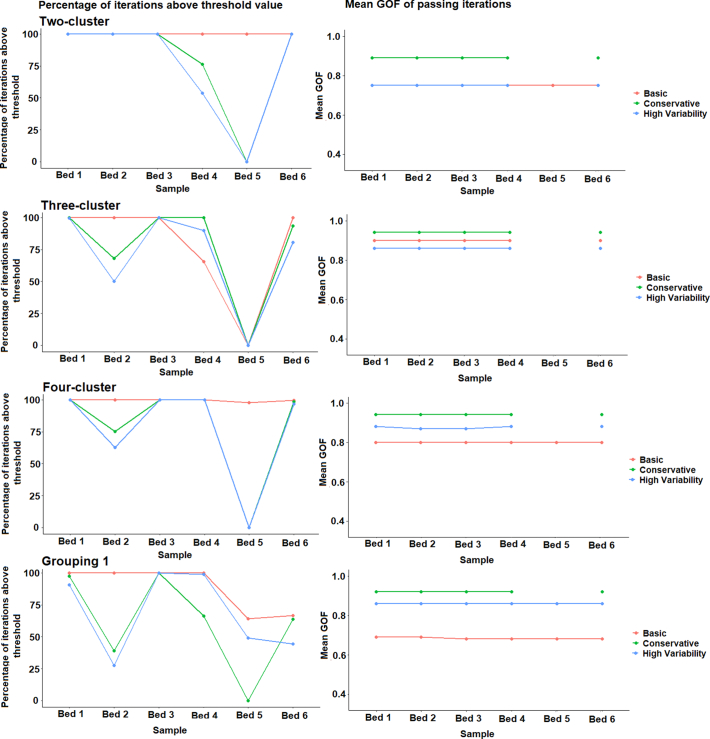
Percentage of Monte Carlo iterations above the 0.35 threshold and mean GOF of iterations passing the threshold.

Supplementary Table 1 The colour (A) and magnetic (B) signatures used, their method of calculation and the property they represent. Colour signatures are based upon their use by Viscarra Rossel et al. (2006) and Ray et al. (2004). Supplementary Table 1

Supplementary figures Image 1
