# Supplementary figures and images for: Tracing catchment fine sediment sources using the new SIFT (SedIment Fingerprinting Tool) open source software
Source: Sci Total Environ. 2018 Sep 1;635:838–58. doi: 10.1016/j.scitotenv.2018.04.126 (PMC6024566; doi:10.1016/j.scitotenv.2018.04.126)

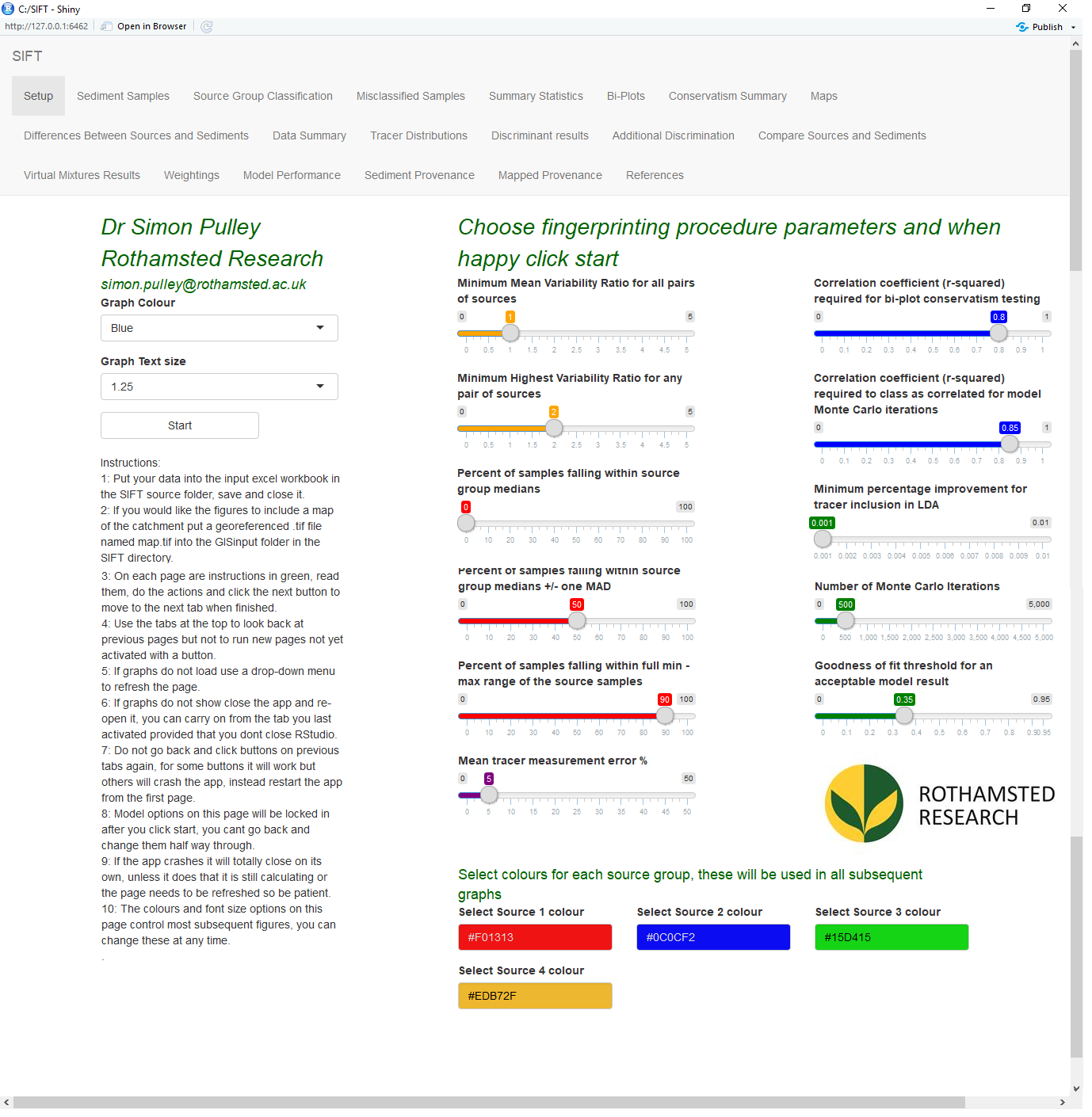

Supplement: Supplementary file 2 — Supplementary figures [file mmc2.zip › SIFT Page 1.tif]

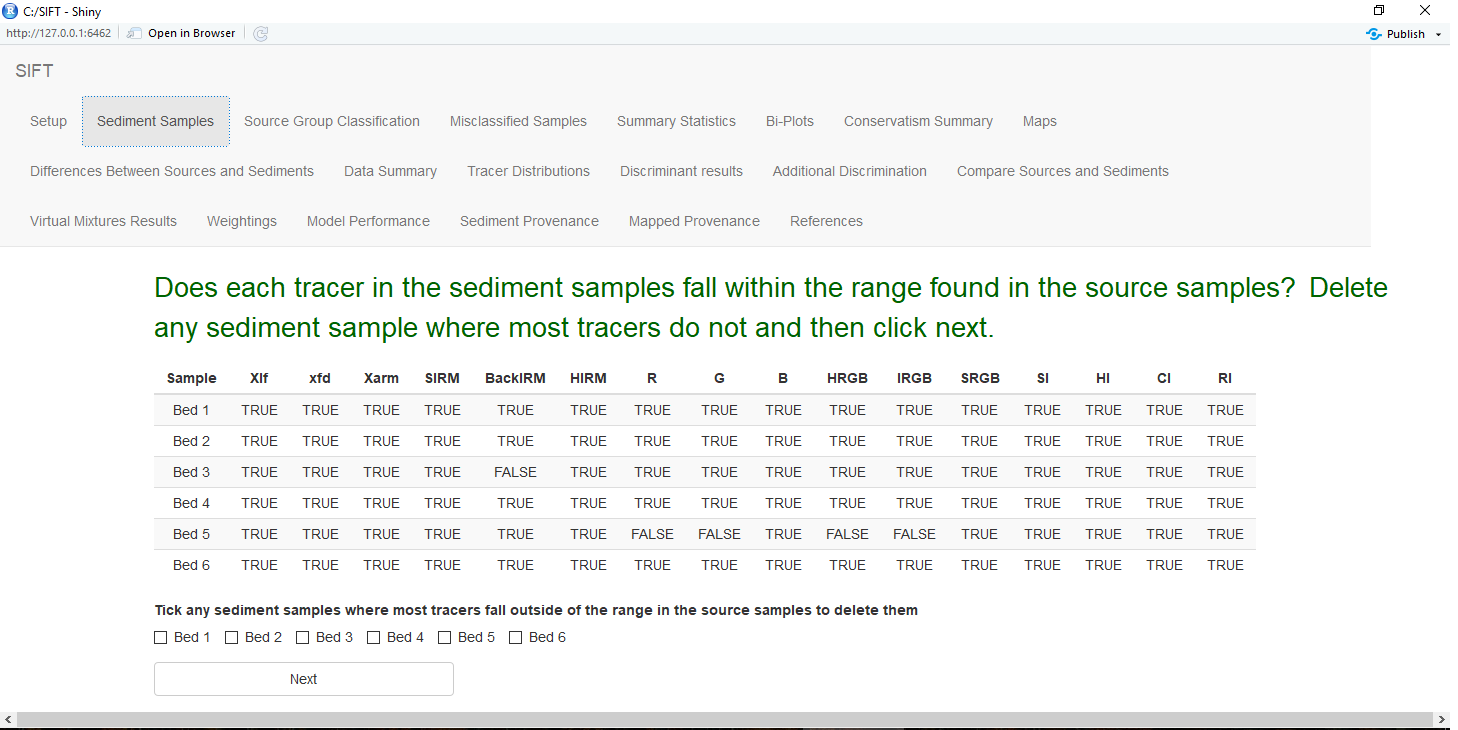

Supplement: Supplementary file 2 — Supplementary figures [file mmc2.zip › SIFT Page 2.tif]

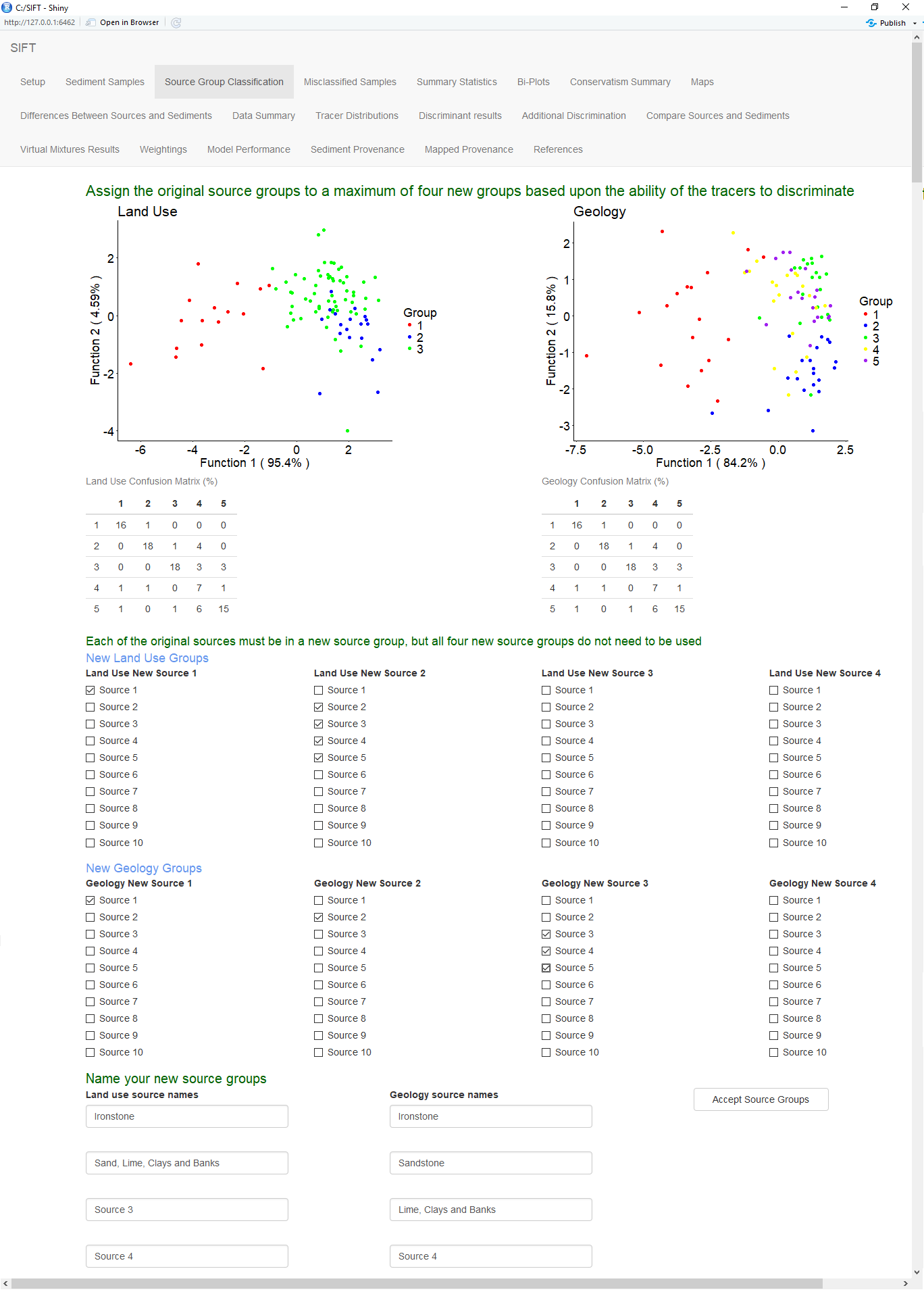

Supplement: Supplementary file 2 — Supplementary figures [file mmc2.zip › SIFT Page 3.tif]

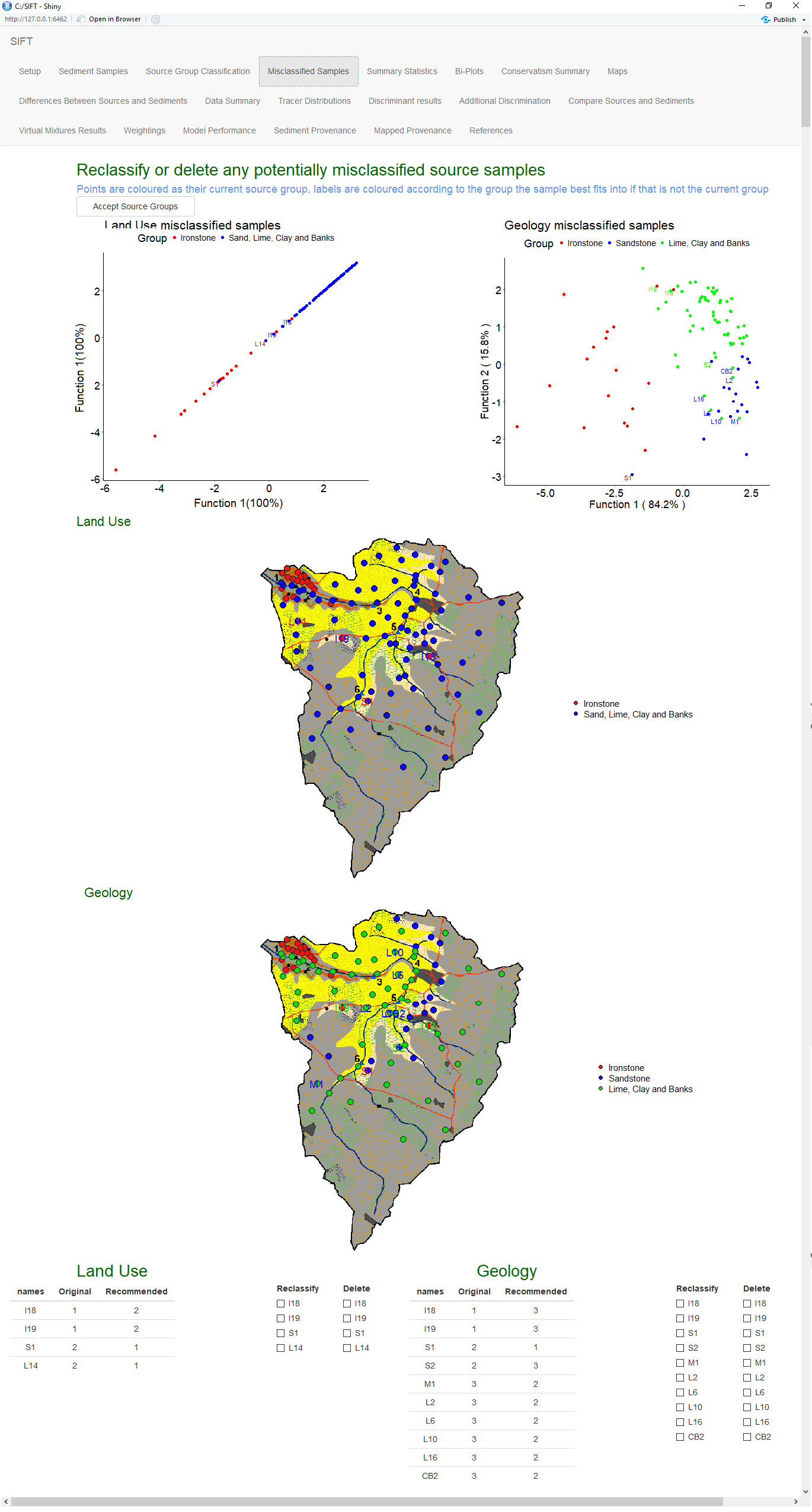

Supplement: Supplementary file 2 — Supplementary figures [file mmc2.zip › SIFT Page 4.tif]

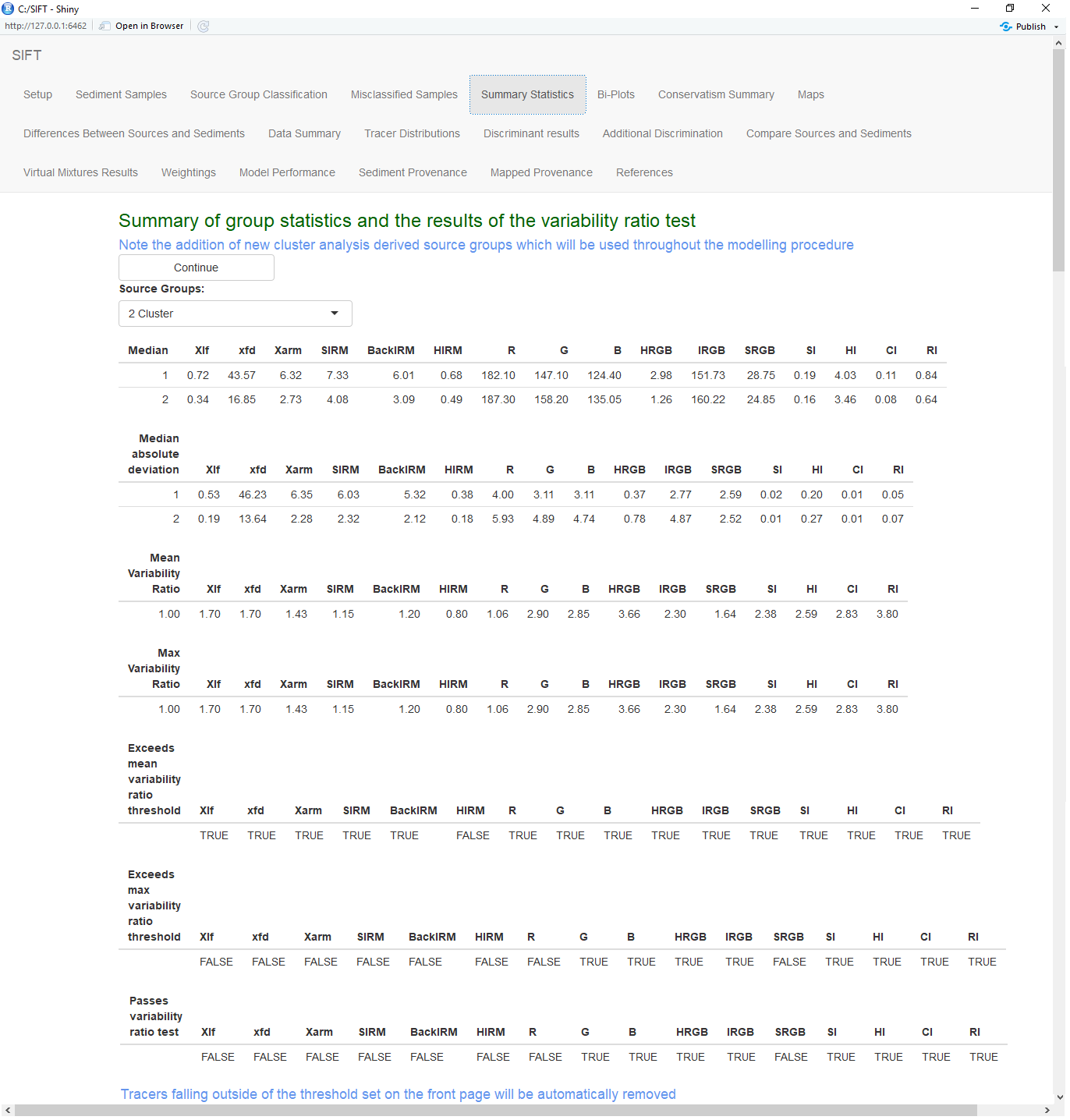

Supplement: Supplementary file 2 — Supplementary figures [file mmc2.zip › SIFT Page 5.tif]

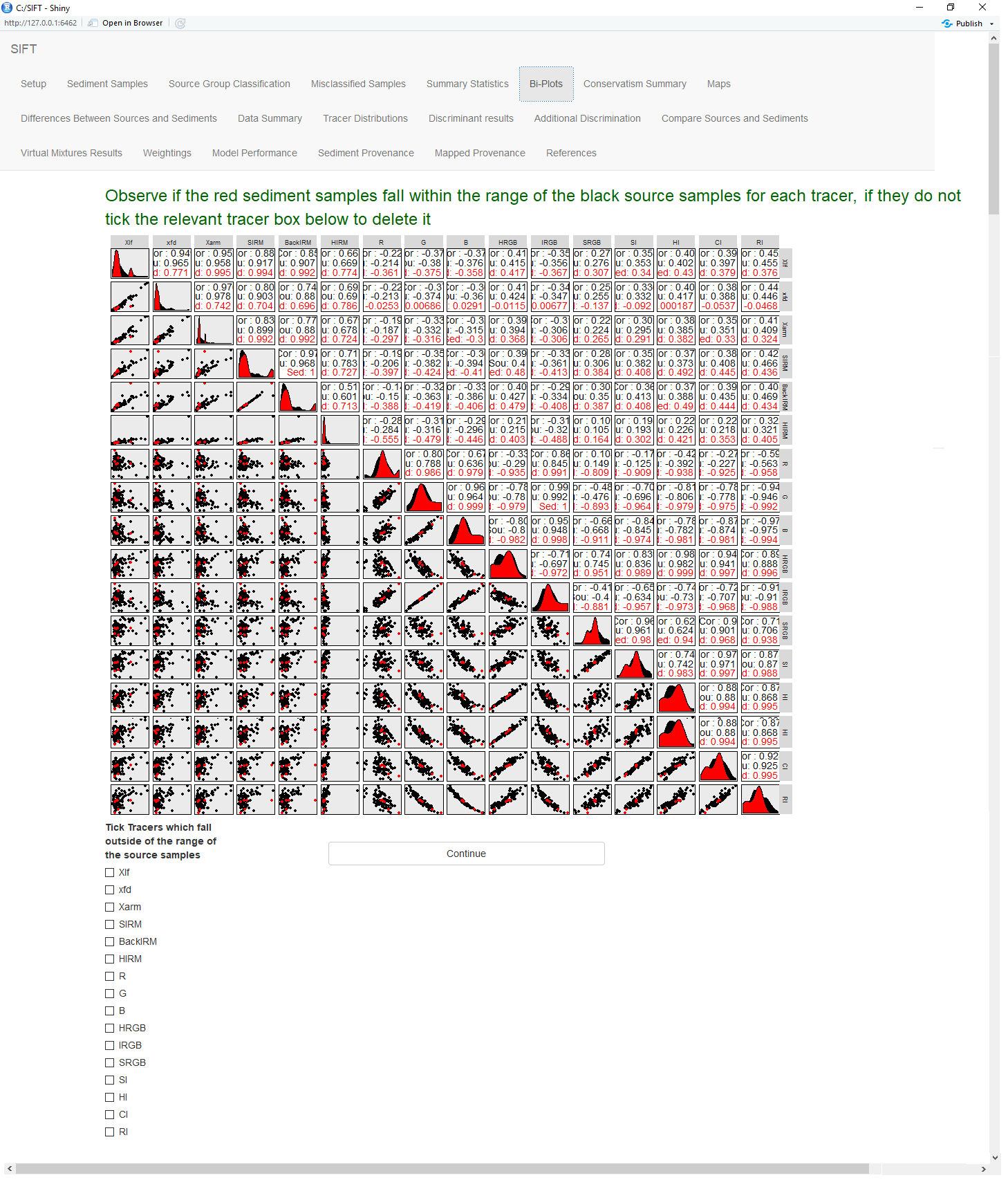

Supplement: Supplementary file 2 — Supplementary figures [file mmc2.zip › SIFT Page 6.tif]

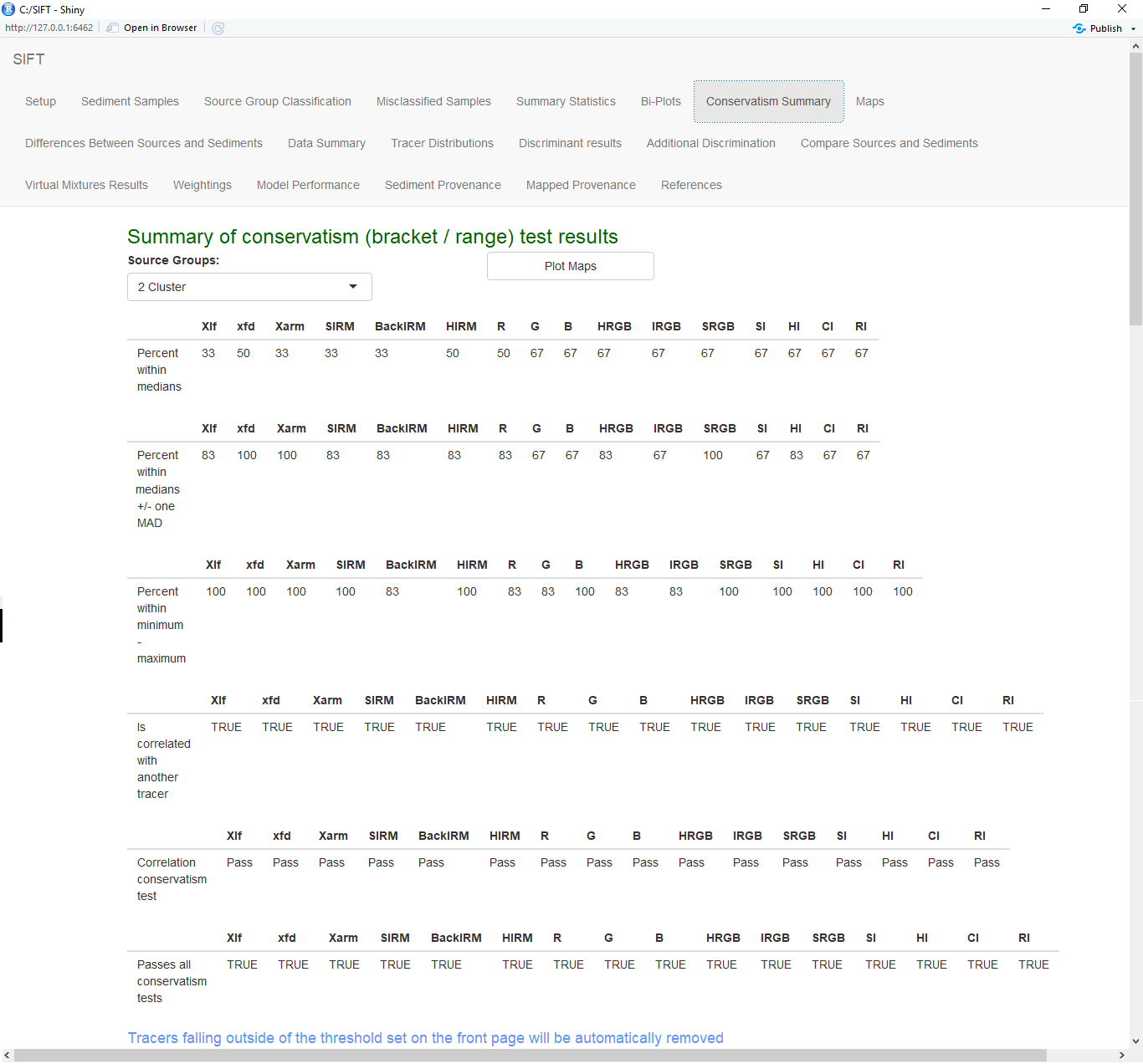

Supplement: Supplementary file 2 — Supplementary figures [file mmc2.zip › SIFT Page 7.tif]

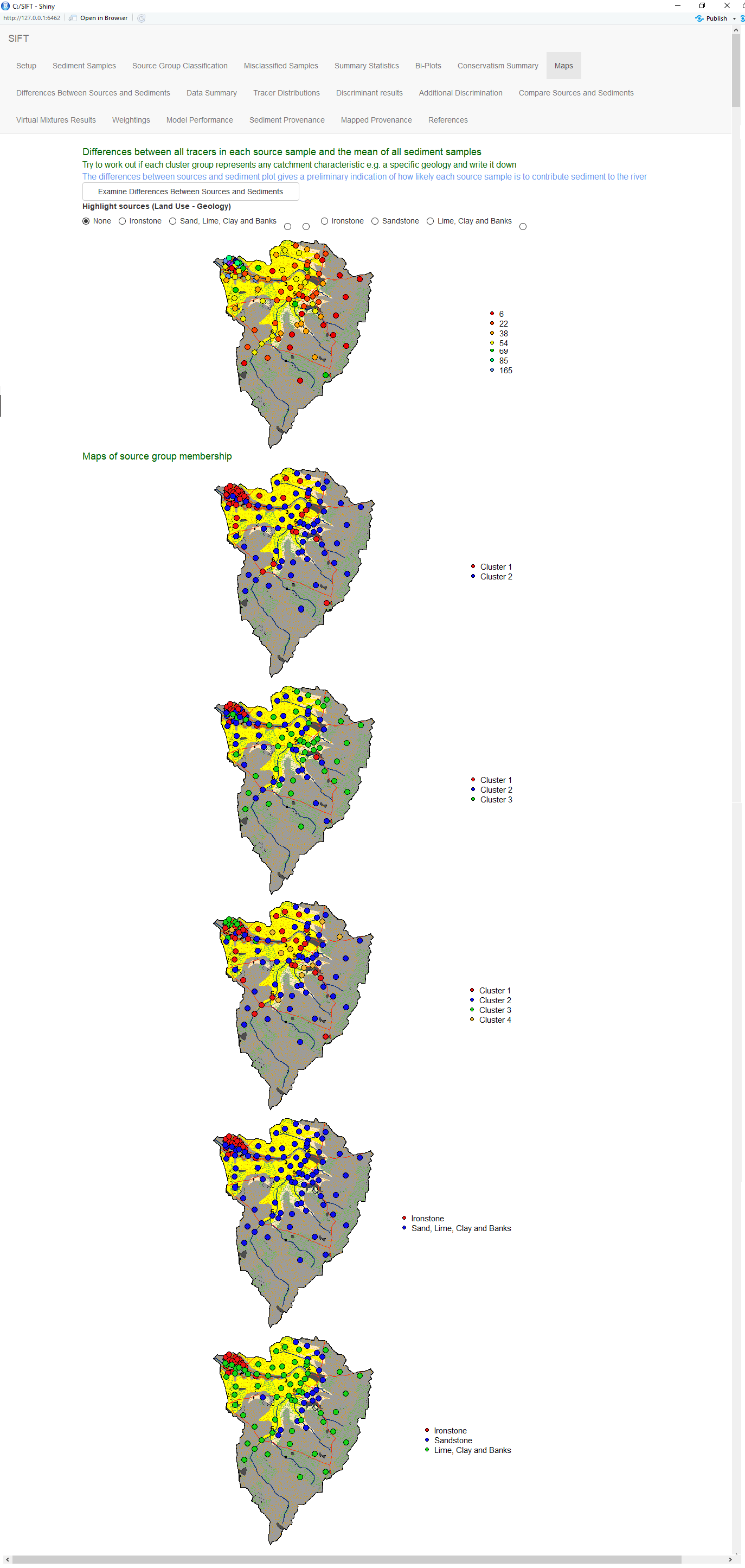

Supplement: Supplementary file 2 — Supplementary figures [file mmc2.zip › SIFT Page 8.tif]

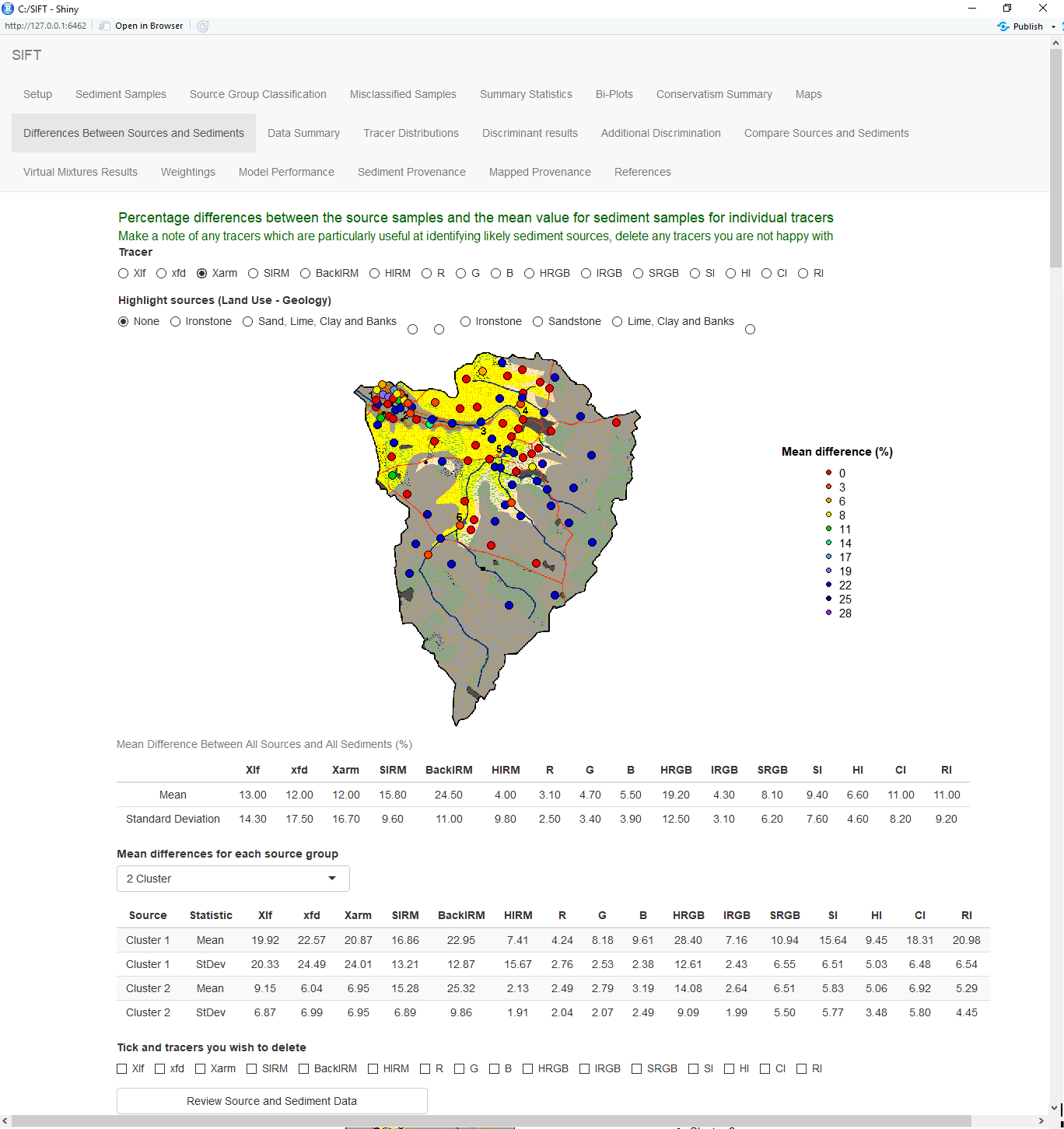

Supplement: Supplementary file 2 — Supplementary figures [file mmc2.zip › SIFT Page 9.tif]

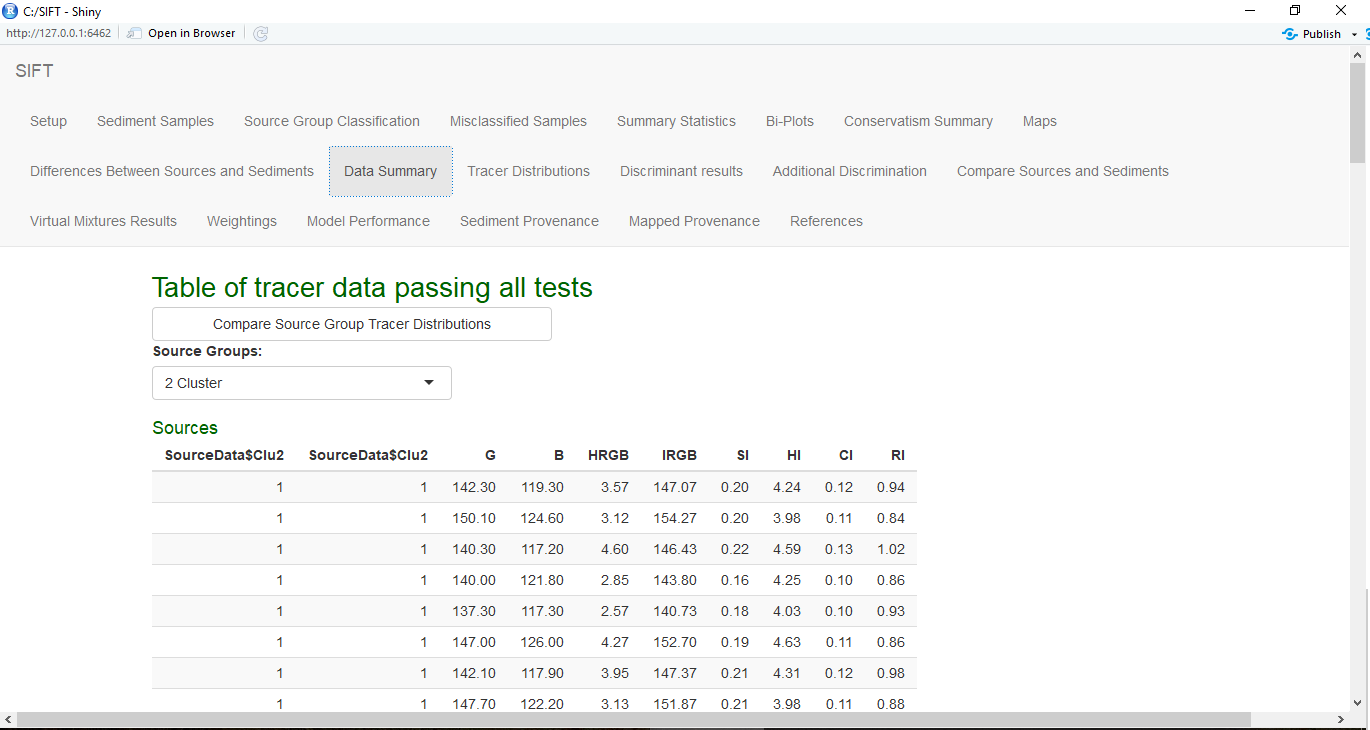

Supplement: Supplementary file 2 — Supplementary figures [file mmc2.zip › SIFT Page 10.tif]

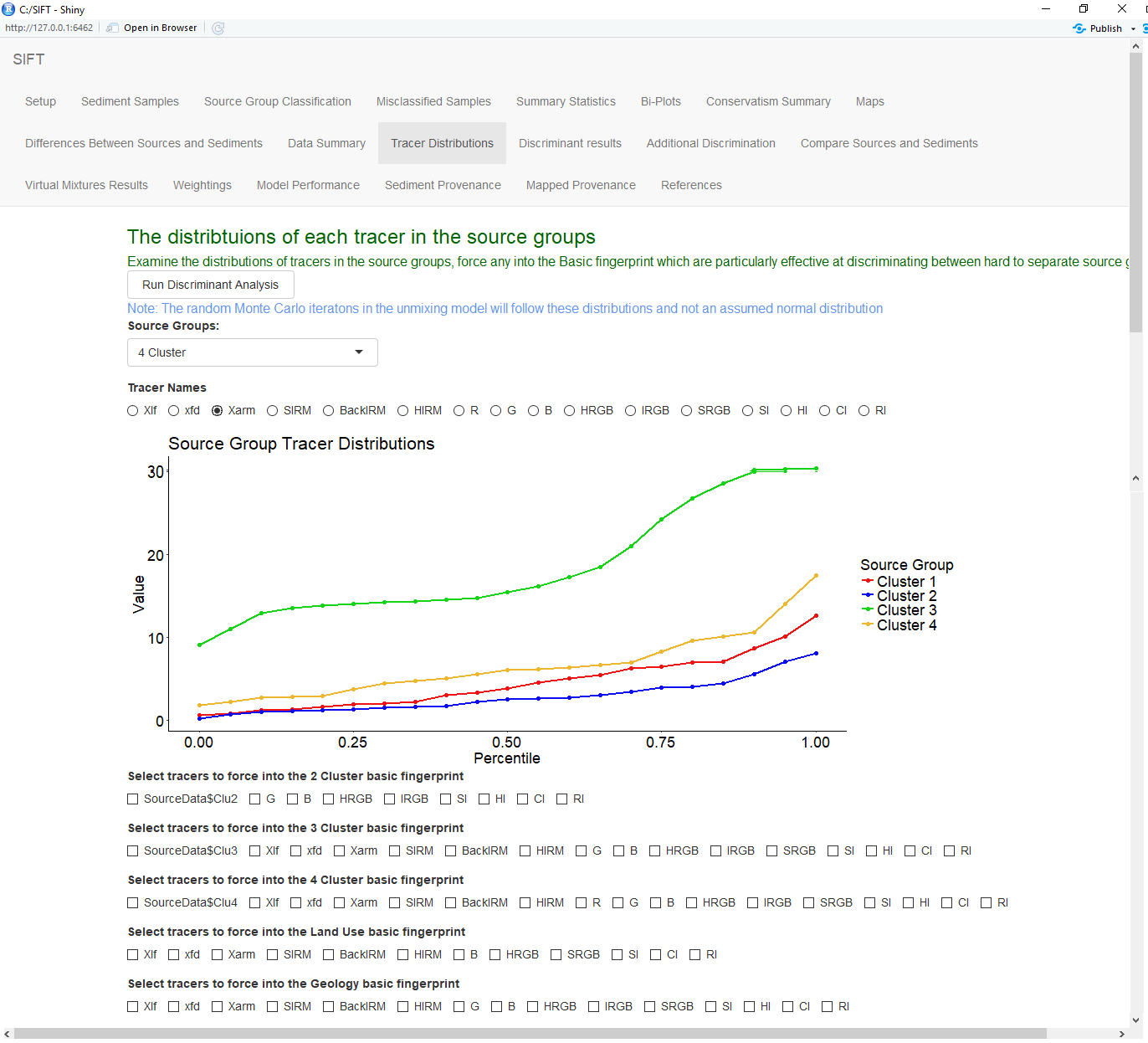

Supplement: Supplementary file 2 — Supplementary figures [file mmc2.zip › SIFT Page 11.tif]

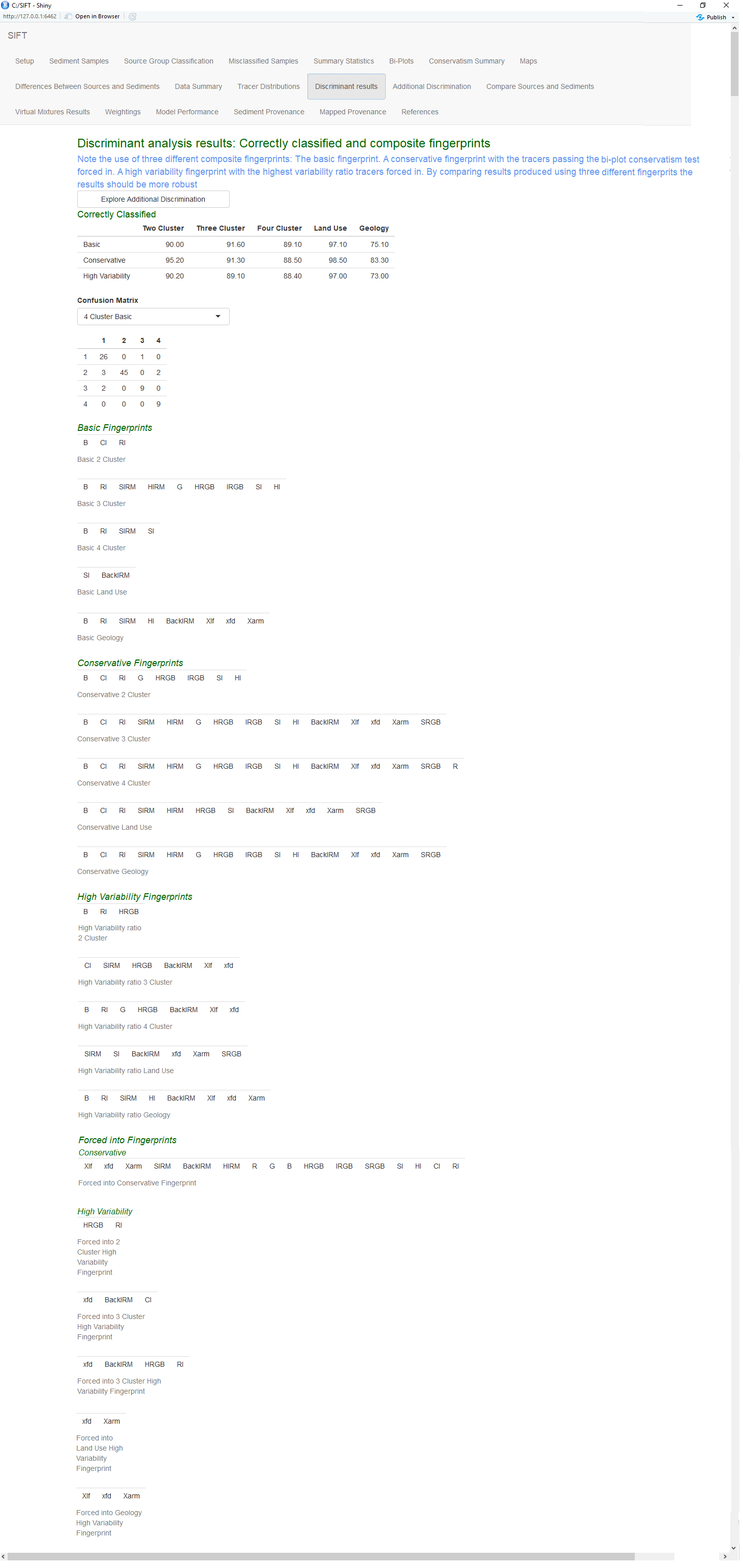

Supplement: Supplementary file 2 — Supplementary figures [file mmc2.zip › SIFT Page 12.tif]

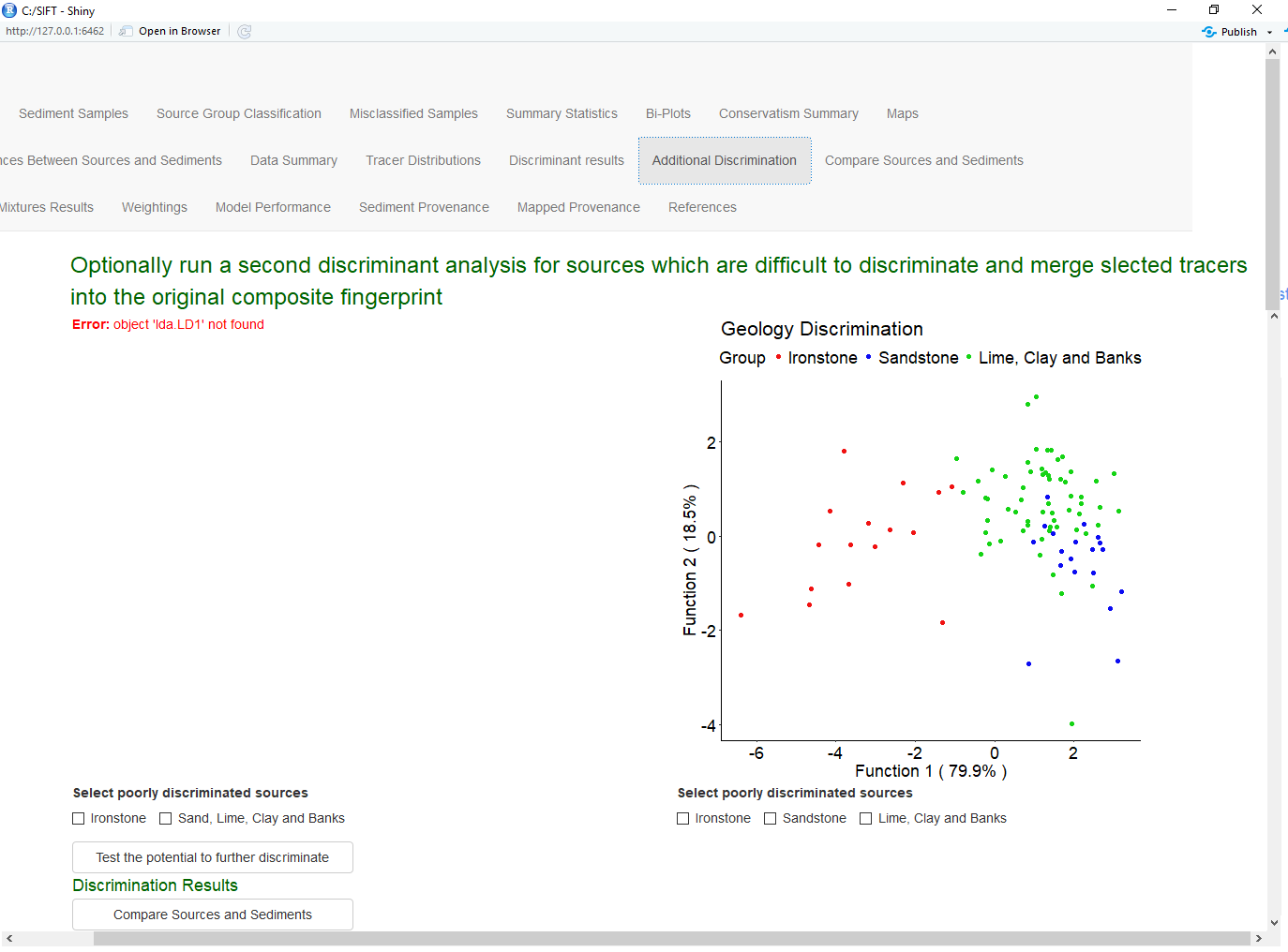

Supplement: Supplementary file 2 — Supplementary figures [file mmc2.zip › SIFT Page 13.tif]

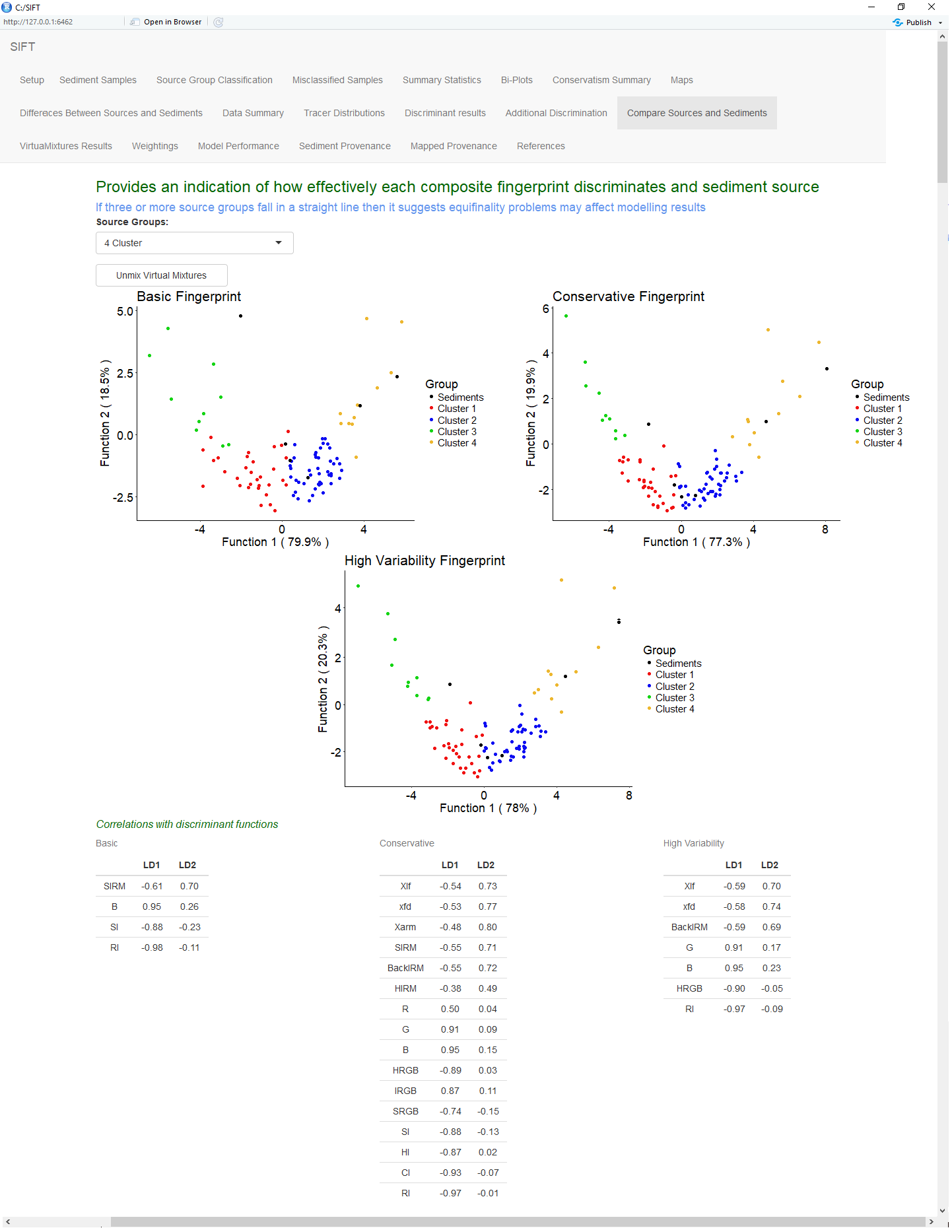

Supplement: Supplementary file 2 — Supplementary figures [file mmc2.zip › SIFT Page 14.tif]

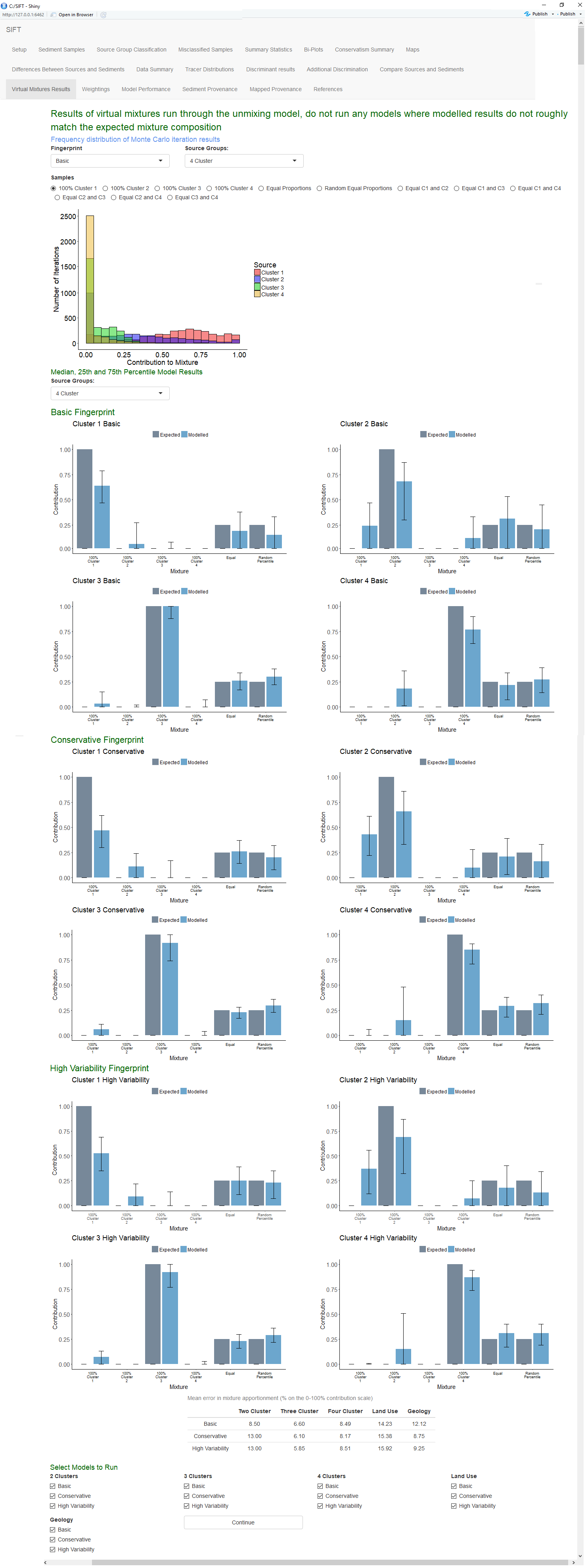

Supplement: Supplementary file 2 — Supplementary figures [file mmc2.zip › SIFT Page 15.tif]

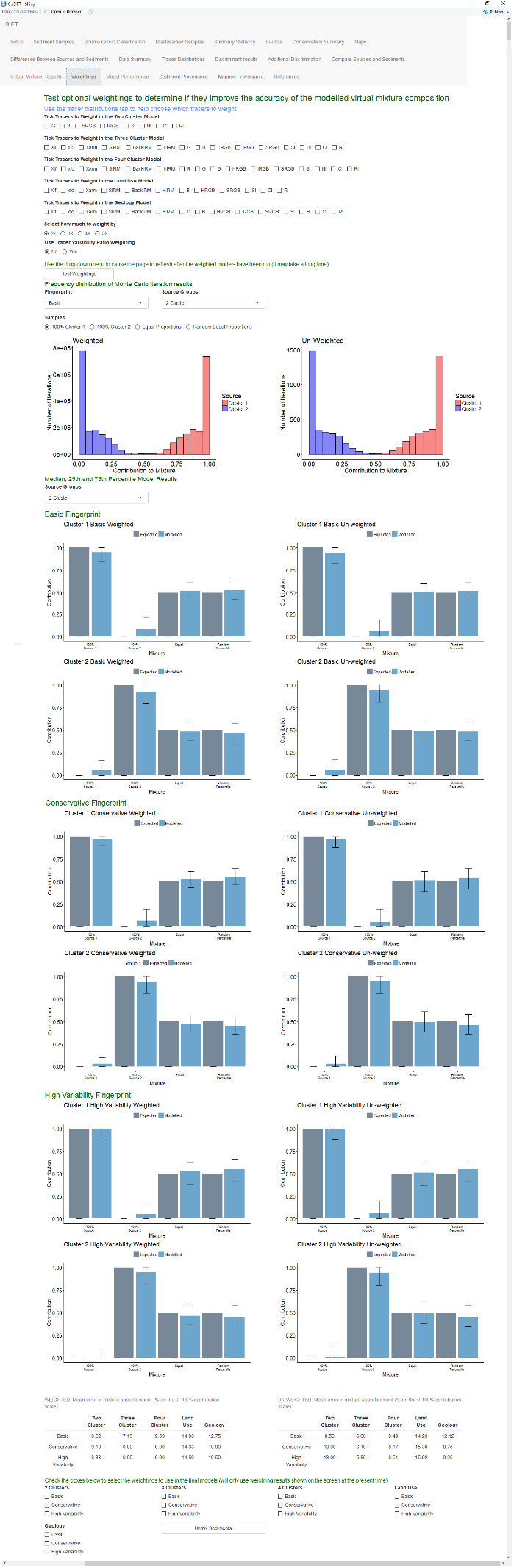

Supplement: Supplementary file 2 — Supplementary figures [file mmc2.zip › SIFT Page 16.tif]

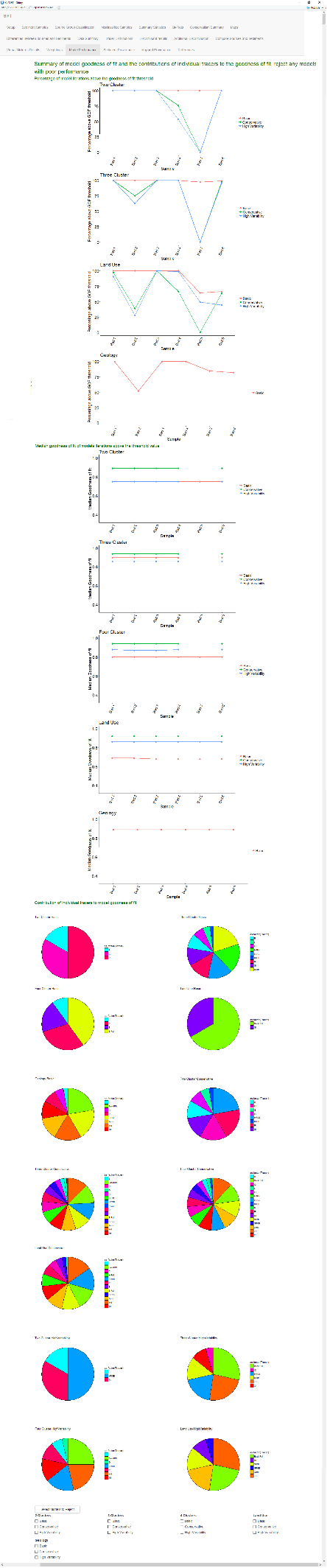

Supplement: Supplementary file 2 — Supplementary figures [file mmc2.zip › SIFT Page 17.tif]

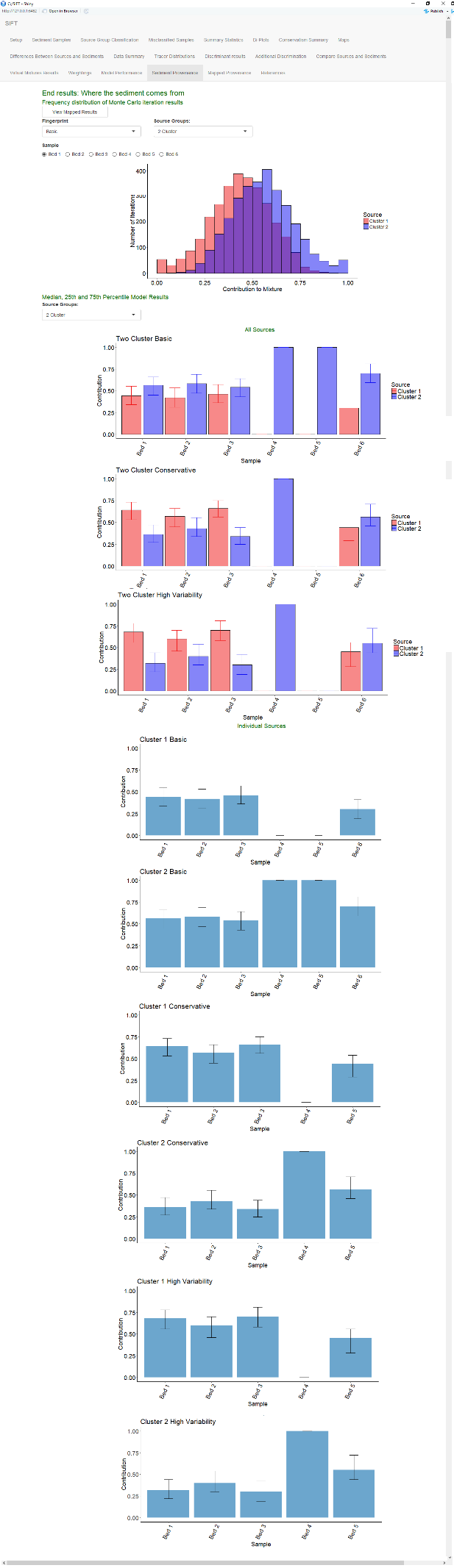

Supplement: Supplementary file 2 — Supplementary figures [file mmc2.zip › SIFT Page 18.tif]

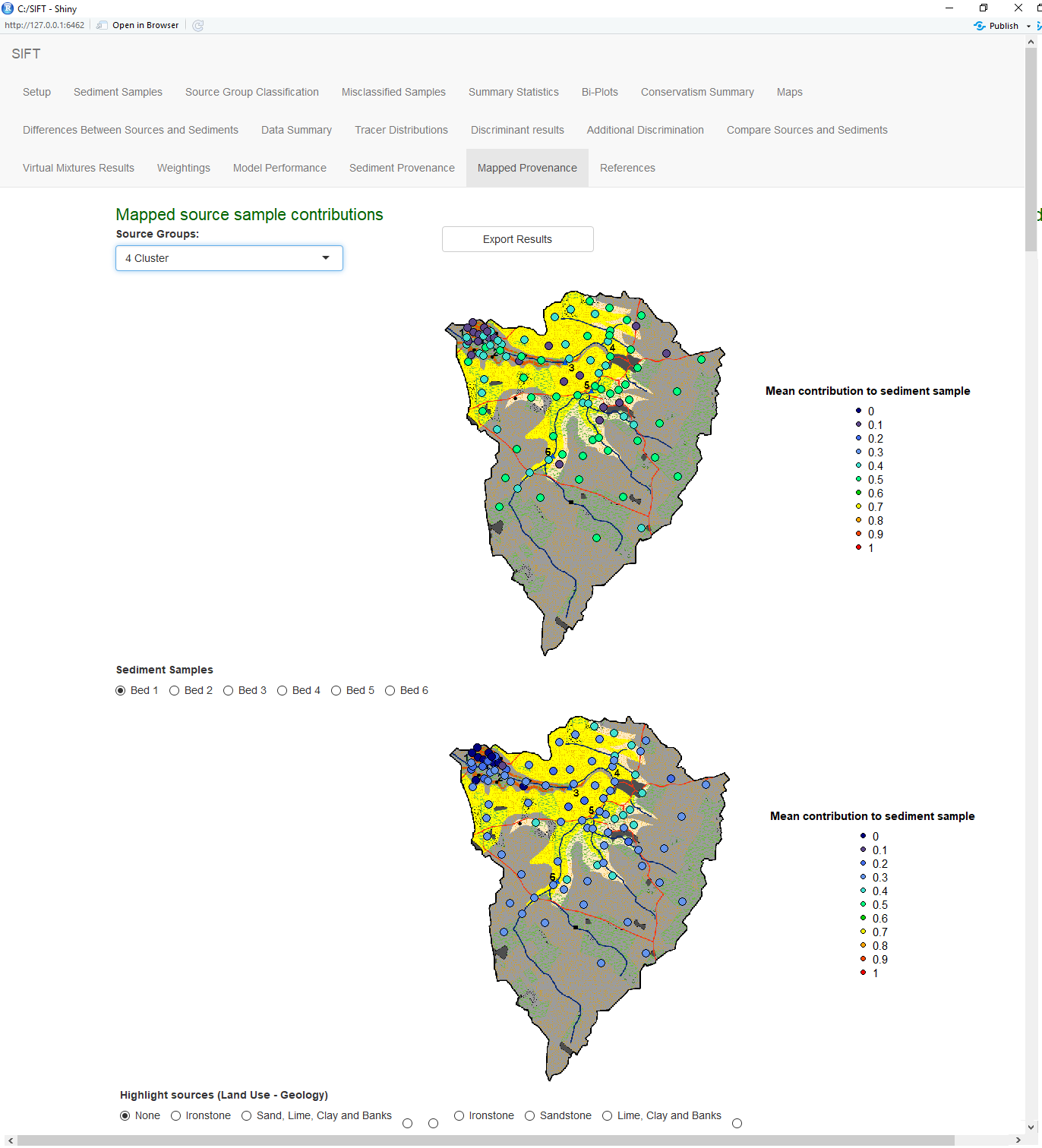

Supplement: Supplementary file 2 — Supplementary figures [file mmc2.zip › SIFT Page 19.tif]
